# Integrating QTL mapping and transcriptomics to decipher the genetic architecture of sterol metabolism in *Brassica napus* L

**DOI:** 10.1093/hr/uhae196

**Published:** 2024-07-24

**Authors:** Yiyi Xiong, Guangyuan Lu, Huaixin Li, Jianjie He, Shipeng Fan, Shuxiang Yan, Liangxiao Zhang, Haibo Jia, Maoteng Li

**Affiliations:** Key Laboratory of Molecular Biophysics of the Ministry of Education, College of Life Science and Technology, Huazhong University of Science and Technology, 1037 Luoxiong Road, Hongshan District, Wuhan 430074, China; College of Biology and Food Engineering, Kechuang 1st Road, Maonan District, Guangdong University of Petrochemical Technology, Maoming 525000, China; Key Laboratory of Molecular Biophysics of the Ministry of Education, College of Life Science and Technology, Huazhong University of Science and Technology, 1037 Luoxiong Road, Hongshan District, Wuhan 430074, China; Key Laboratory of Molecular Biophysics of the Ministry of Education, College of Life Science and Technology, Huazhong University of Science and Technology, 1037 Luoxiong Road, Hongshan District, Wuhan 430074, China; Key Laboratory of Molecular Biophysics of the Ministry of Education, College of Life Science and Technology, Huazhong University of Science and Technology, 1037 Luoxiong Road, Hongshan District, Wuhan 430074, China; Key Laboratory of Molecular Biophysics of the Ministry of Education, College of Life Science and Technology, Huazhong University of Science and Technology, 1037 Luoxiong Road, Hongshan District, Wuhan 430074, China; Key Laboratory of Biology and Genetic Improvement of Oil Crops, Ministry of Agriculture and Rural Affairs, Oil Crops Research Institute, Chinese Academy of Agricultural Sciences, Xudong 2nd Road, Wuchang District, Wuhan 430062, China; Key Laboratory of Molecular Biophysics of the Ministry of Education, College of Life Science and Technology, Huazhong University of Science and Technology, 1037 Luoxiong Road, Hongshan District, Wuhan 430074, China; Key Laboratory of Molecular Biophysics of the Ministry of Education, College of Life Science and Technology, Huazhong University of Science and Technology, 1037 Luoxiong Road, Hongshan District, Wuhan 430074, China

## Abstract

Sterols are secondary metabolites commonly found in rapeseed that play crucial physiological roles in plants and also benefit human health. Consequently, unraveling the genetic basis of sterol synthesis in rapeseed is highly important. In this study, 21 individual sterols as well as total sterol (TS) content were detected in a double haploid (DH) population of *Brassica napus*, and a total of 24 quantitative trait loci (QTL) and 157 mQTL were identified that were associated with TS and different individual sterols. Time-series transcriptomic analysis showed that the differentially expressed genes (DEGs) involved in sterol and lipid biosynthesis pathways were enriched. Additionally, a regulatory network between sterol-related DEGs and transcription factors (TFs) was established using coexpression analysis. Some candidate genes were identified with the integration of transcriptomic analysis and QTL mapping, and the key candidate gene *BnSQS1*.*C03* was selected for further functional analysis. *BnSQS1.C03* demonstrated squalene synthase activity *in vitro* and increased the TS by 3.8% when overexpressed in *Arabidopsis*. The present results provide new insights into sterol regulatory pathways and a valuable genetic basis for breeding rapeseed varieties with high sterol content in the future.

## Introduction

*Brassica napus* L. (AACC, 2*n* = 38) is of great significance in the food and chemical industries [1], with ~70 million tons of rapeseed produced annually worldwide [2]. Previous studies have revealed that most agronomic characteristics of *B. napus* are quantitative traits, and their genetic mechanisms have frequently been studied using QTL analysis [3–5]. In addition to the genetic improvement of agronomic traits, the genetic enhancement of secondary metabolites, such as vitamins, glucosinolates, carotenoids, and phytosterols, has been increasingly garnering attention in recent years, particularly in *B. napus*, due to their health-promoting properties [6, 7].

Studies have shown that phytosterols can decrease low-density lipoprotein cholesterol and change sterol formation of human body [8, 9]. Phytosterols have also been shown to have anti-inflammatory [10, 11], cytoprotective [11], and cardiovascular protective effects [12]. Some special sterols have been confirmed to have significant health effects. For example, squalene is commonly used as an auxiliary medicine and as a natural cardiovascular protection drug [13, 14]. The antitumor activity of phytosterols have also been reported. For example, brassicasterol, a specialized sterol in rapeseed, has been shown to possess antiviral and anticancer functionalities [15]. Phytosterols are present in vegetable oils with varied concentrations and compositions and have been detected in rice bran oil, corn oil, and rapeseed oil [16, 17]. Therefore, increasing the phytosterol content might represent a new method to enhance the quality of edible oils [18, 19]. Phytosterols also have important physiological functions in plants; for example, they are important components of membrane structures [20, 21]. Sterols are precursors for brassinosteroid (BR) synthesis [22], and some sterols can also produce BR-independent effects [23, 24].

A correlation between sterol synthesis and lipid synthesis has been reported, revealing that the sterol upstream synthesis genes 3-hydroxy-3-methylglutaryl-coenzyme A reductase 1 (*HMG1*) and acetyl-CoA carboxylase 1 (*ACC1*) are coregulated by adenosine 5′-monophosphate (AMP)-activated protein kinase (*AMPK*) [25]. The overexpression of fatty acid synthesis-related genes (including *ACC1*) can induce the expression of genes involved in several sterol biosynthetic pathways, resulting in increased levels of multiple sterols [26]. In addition, sterol is also involved in oil body formation, lipoglobulin synthesis, and lipid accumulation due to the presence of sterols in the membrane structure, which form lipid rafts and sterol ester vesicles on the membrane [27]. Genetic molecular analyses of sterols were performed in previous studies. For example, 45 QTL associated with sterol content in sunflower seeds were identified, and these genes were distributed in 9 linkage groups. In maize, 9 SNPs on 5 chromosomes were identified as associated with total sterols (TSs) and some sterol components using GWAS analysis [28, 29]. Although sterols have been reported in *B. napus* for a long time [30], few studies have been conducted to elucidate the underlying genetic mechanism at the population level. In 2008, a population containing 148 double haploid (DH) lines was used for QTL mapping of four phytosterols and TS, and 27 QTL were successfully identified [31]. Teh and Möllers used a population that consisted of 226 DH lines to perform QTL analysis for the TS and four individual sterols, and 13 QTL associated with four types of phytosterols and two QTL of the TS were identified [32].

Metabolomics is an efficient omics tool that has been widely used in crop improvement [33–35]. For previous quantitative sterol determination methods, the samples require saponification, purification, and concentration steps before determination, which necessitates considerable work, time, and materials [31]. Moreover, due to the detection and quantification limits of the methods, the detected sterol species and the accuracy of the determined content are restricted [36]. The latest method for sterol detection in food sources using MS/MS has reduced the complexity of sample preparation, and both free and bound sterols can be detected simultaneously [37].

In recent years, metabolomics combined with QTL, genome-wide association study (GWAS), and transcriptomics have frequently been used to discover the genetic mechanism of plant metabolite changes [38]. For example, the metabolites in different tissues of 282 maize inbred lines were analyzed using GWAS analysis as well as by mapping isomeric variation, and a single linkage block in a citrate synthase-like gene was found [39]. In loquat, the candidate genomic regions associated with fruit weight were identified using a combination of QTL mapping, transcriptome profiling, and metabolic profiling [40]. By utilizing metabolomics on 388 lines, 2172 metabolites were quantified in *B. napus*, revealing that 131 marker metabolites were correlated with seed oil content (SOC), and *BnaC05*.*UK* was identified as a novel negative regulator of SOC based on the integration of mGWAS, mTWAS, and eGWAS data [41].

In this study, QTL analysis was performed on the 21 important components in the biosynthetic pathway of sterols and TS using a KN DH population constructed by Wang *et al*. [42]. In addition, the DEGs were screened using transcriptomic data obtained from materials with high and low TS in the KN DH population at different seed developmental stages. Based on the QTL mapping and RNA-seq data, a potential regulatory model was established to explain the variation in sterol content. Finally, based on a combined analysis of QTL and RNA-seq data, the key gene *BnSQS1.C03* that simultaneously affects both TS and SOC was identified, and its function was analyzed. The present study provides new insights into the genetic and metabolic controls of sterol content.

## Results

### Variation in sterols and their correlations in *B. napus*

**Figure 1:**
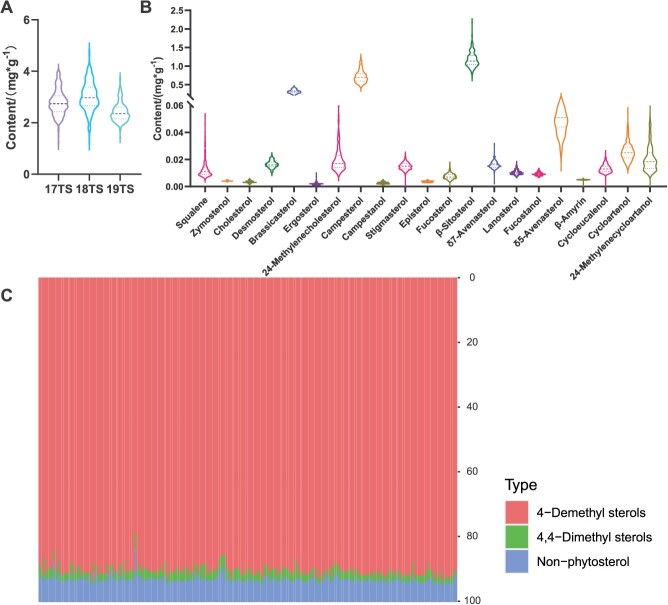
Frequency distribution of sterol content in the KN DH population. (A, B) The TS over 3 years and the content of 21 individual sterols. The top and bottom two of the three dashed lines indicate the quartiles, and the middle dashed line indicates the median. (C) Proportions of different types of sterols in rapeseeds; different colors indicate different types of sterols.

The TS in the seeds of the KN DH population harvested in 2017, 2018, and 2019 ranged from 2.402 to 4.013 mg/g, from 2.365 to 4.787 mg/g and from 2.594 to 3.743 mg/g, respectively (Table S1). All the variables exhibited a normal or near-normal distribution in the KN DH population (Fig. 1A).

The contents of 21 individual phytosterol-related components were also measured in the seeds harvested in 2019 (Table S1). All the components exhibited a normal or near-normal distribution in the KN DH population, and obvious transgressive segregation was also observed (Fig. 1B). Further analysis revealed that the most abundant compounds were β-sitosterol (0.704–2.177 mg/g), campesterol (0.408–1.234 mg/g), and brassicasterol (0.159–0.458 mg/g). However, the contents of other components, such as campestanol (0.001–0.005 mg/g), β-armyrin (0.001–0.006 mg/g), and zymostenol (0.003–0.005 mg/g), were relatively low (<0.1 mg/g) (Table S1). Among the 21 individuals, seven were phytosterols, including four 4-demethyl sterols (β-sitosterol, stigmasterol, campesterol, and brassicasterol) and three 4,4-dimethyl sterols (cycloartanol, cycloartenol, and 24-methylenecycloartanol). 4-Demethyl sterols constitute the largest proportion (78.92–93.65%) of TS in rapeseeds (Fig. 1C). In contrast, the three 4,4-dimethyl sterols and the remaining fourteen nonphytosterols accounted for only 0.97–7.74% and 5.16–16.49%, respectively.

Correlation analysis between the 21 individual components and TS in 2019 revealed that 12 of the individual components were significantly positively correlated with TS, including the three most abundant components (Fig. S1, Table S2). For example, the correlation coefficients between TS and campesterol and between TS and β-sitosterol were 0.864 and 0.856 (*P* < 0.01), respectively. Cluster analysis of these 22 data sets revealed three distinct groups (Fig. S1). Specifically, cycloartenol, squalene, 24-methylenencycloartanol, and cycloeucalenol were classified into one cluster, and three of these components showed a significant negative correlation with TS. For instance, the correlation coefficients between TS and 24-methylenencycloartanol and between TS and cycloeucalenol were −0.290 and −0.264 (*P* < 0.01), respectively.

A correlation between SOC and TS, as well as their different compositions, was also detected (Fig. S2, Table S2), and no significant correlation between TS and SOC was found. In contrast, four compounds (cycloartenol, squalene, 24-methylenecycloartenol, and cycloeucalenol) showed a highly significant positive correlation (*P* < 0.01) with SOC, and cycloeucalenol had a correlation coefficient of 0.558. Moreover, some sterols showed a significant negative correlation with SOC (β-sitosterol, stigmastanol, etc.), but the correlation coefficient was relatively low.

### QTL mapping of TS and individual sterols

**Figure 2:**
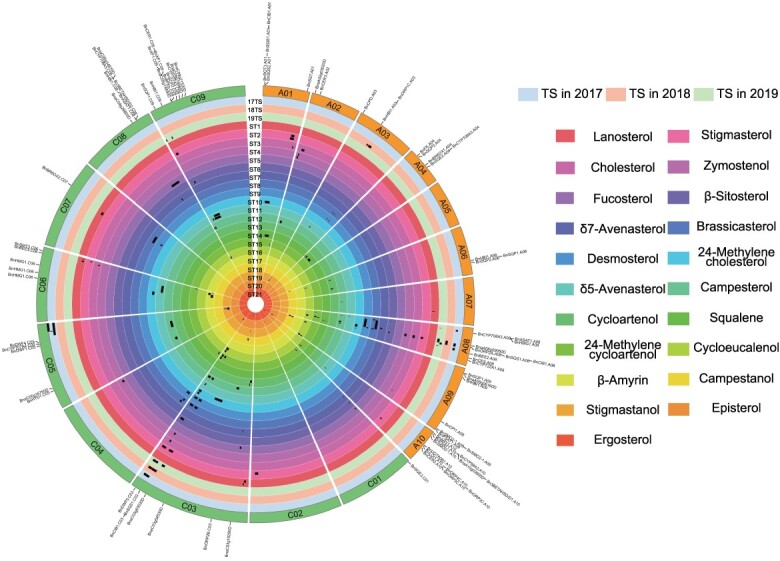
The distribution of all QTL for sterols on 19 chromosomes and the related gene annotations within the intervals. The outermost layer is the name of the candidate gene within the QTL interval. The inward layer is the physical length of chromosomes. In total, 21 individual sterols as well as TS levels measured over the 3 years are reported in the innermost circle, and the traits corresponding to the different circles are listed on the right. The TS are indicated with distinct symbols on the outer side. The black rectangles indicate the different QTL intervals.

QTL mapping of 21 individual components and TS was performed using a high-density linkage map of the KN DH population that was constructed previously. In total, 157 mQTL for 21 sterol components and 24 QTL for TS were identified (Fig. 2, Table 1, Table S3).

These 24 TS were distributed on chromosomes A03, A08, C03, C05, and C09 of *B. napus*, with QTL numbers of 2, 8, 6, 6, and 2, respectively. Further analysis revealed that only QTL that generated in 2017 were detected on C05, and the phenotypic variation (PV) of these QTL was relatively low (5.133–6.001%). In contrast, the QTL detected in three successive years were mainly located on A08 and C03, and these QTL also had relatively greater PVs (4.446–17.283%). Further analysis revealed that the 24 QTL could be integrated into 17 consensus QTL, and two consensus QTL (*cqTS-A08-2* and *cqTS-C03-2*) and three QTL (*cqTS-A08-1*, *cqTS-A08-4*, and cq*TS*-C03-3) were colocalized with 3 and 2 years, respectively.

The 157 mQTL were distributed across all 19 chromosomes of *B. napus*, with the number of mQTL on 10 chromosomes (A02, A04, A05, A06, A07, A10, C02, C05, C07, and C08) being less than 5. On the remaining nine chromosomes, the number of mQTL was not less than 7 each. Further analysis revealed that A08 and C03 had the most mQTL, at 26 and 38, respectively. A large number of hotspot regions that simultaneously influence the content of multiple individual sterols were also identified (Fig. S3A). For a single component, campesterol had the most mQTL (13 mQTL), whereas 2 mQTL were obtained for ergosterol. Among all mQTL, the four adjacent mQTL for squalene content (*qST14-A04-1*, *qST14-A04-2*, *qST14-A04-3*, and *qST14-A04-4*) exhibited high PVs of 22.2138%, 27.0219%, 32.4779%, and 30.7315%, respectively (Fig. S3B). *qST18-C03–1* and *qST18-C03-2*, which are located on chromosome C03 and are associated with campestanol content, also exhibited high PVs of 21.1079% and 27.1765%, respectively (Fig. S3C, D).

### Colocalization analysis of TS QTL and mQTL for single sterols and SOC QTL

Colocalization analysis of mQTL with TS QTL were performed and revealed that 21 mQTL colocalized with the TS QTL on A08, and *cq-TS-A08-2* overlapped with the most mQTL (9 mQTL). Regarding C03, 23 mQTL colocalized with TS QTL, and *cq-TS-C03-2* overlapped with most mQTL (12 mQTL). In contrast, only four mQTL that colocalized with the TS QTL were found on C09. Further analysis revealed that most of these overlapping regions were located in hotspot regions on A08 and C03, and mQTL of the three most abundant components (β-sitosterol, brassicasterol, and campesterol) exhibited the most overlapping regions with the TS QTL. These loci contained several loci with high PV, such as *qST6-C03-5*, *qST8-C03-7*, *qST12-A08-4*, and *qST12-C03-8*, which had PVs of 12.79%, 13.97%, 15.93%, and 13.58%, respectively.

The colocalization of QTL for SOC with TS QTL and mQTL was also analyzed. The SOC was measured in 2019, and QTL analysis was conducted (Table S4). The QTL for SOC located on A08 (for example, *qSOC-A08-1, qSOC*-*A08*-*2*, and *qSOC-A08-3*) and C03 (for example, *qSOC-C03-1*, *qSOC-C03-2*, and *qSOC-C03-3*) were colocalized with QTL hotspot regions of sterol (Fig. S4). In addition, some TS QTL and mQTL were also localized to similar confidence intervals (CIs) with SOC on chromosomes A08 and C03, including *q17TS-A08-1*, *q19TS-C03-1*, *qST10-A08-1*, and *qST11-C03-1*. According to the phenotypic analysis, among the 21 individual components, cycloeucalenol was significantly correlated with SOC. According to the QTL colocalization analysis, a greater number of QTL were colocalized with SOC. For example, *qST16-A08-1* was colocalized with *qSOC-A08-1*, and *qST16-C03-1* and *qST16-C03-2* were colocalized with *qSOC-C03–2*. The mQTL for 24-methylencycloartenol also demonstrated a strong association with SOC QTL, for instance, *qST15-A08-2* and *qST15-A08-3* with *qSOC-A08-1* as well as *qST15-C03-1* and *qST15-C03-2* with *qSOC-C03-2*. The TS QTL could also be colocalized in multiple CIs of the QTL for SOC, but this colocalization was not significant.

### Detection of candidate genes in confidence intervals of QTL

The candidate genes in all CIs of TS QTL and mQTL were predicted by the covariance between genetic and physical mapping. Moreover, sterol-related genes that were clearly reported in the model plant *A. thaliana* were also obtained from querying databases. A total of 97 and 254 sterol-related genes in the CIs of the TS QTL and mQTL regions were obtained, respectively (Table S5, Table S6).

**Table 1:** Summary statistics of significant QTL (LOD score > 2.5) detected for 21 individual sterols and TS over the three-year period

QTL	Data	Chromosome	Peak (cM)	Confidence interval (cM)	LOD	*R^2^*	Addictive effect	Genomic region (Mb)	Consensus QTL
*q18TS-A03–1*	18TS	A03	65.51	64.7–66.3	4.36387	6.1775	0.1568	11.15–11.63	*cqTS-A03–1*
*q18TS-A03–2*	18TS	A03	74.61	74.3–75.8	3.09442	4.4258	0.1322	12.22–14.00	*cqTS-A03–2*
*q19TS-A08–1*	19TS	A08	14.91	12.6–16.2	10.5614	13.69	−0.1412	2.01–2.02	*cqTS-A08–1*
*q17TS-A08–1*	17TS	A08	17.81	13.3–18.1	5.16894	7.2903	−0.1424	2.01–2.69	
*q19TS-A08–2*	19TS	A08	22.01	19.8–24.4	12.7336	15.654	−0.1519	8.37–10.37	*cqTS-A08–2*
*q18TS-A08–1*	18TS	A08	23.01	21.2–25.6	3.21594	4.5361	−0.1273	8.71–10.84	
*q17TS-A08–2*	17TS	A08	24.61	21.4–25.6	7.32158	10.136	−0.1682	9.30–10.84	
*q19TS-A08–3*	19TS	A08	28.01	27.6–30.3	13.3238	17.234	−0.1571	10.37–11.29	*cqTS-A08–3*
*q19TS-A08–4*	19TS	A08	33.31	33–33.5	6.48396	8.1215	−0.1072	12.22–12.35	*cqTS-A08–4*
*q17TS-A08–3*	17TS	A08	33.91	32.3–35.6	4.42463	6.2915	−0.1315	11.70–12.82	
*q19TS-C03–1*	19TS	C03	174.81	173.2–176.1	6.96136	9.0279	−0.123	50.80–52.63	*cqTS-C03–1*
*q18TS-C03–1*	18TS	C03	181.01	179.8–184.3	4.56134	6.5171	−0.161	53.46–56.38	*cqTS-C03–2*
*q19TS-C03–2*	19TS	C03	183.61	182.9–183.7	14.4197	17.283	−0.1769	55.45–56.38	
*q17TS-C03–1*	17TS	C03	185.51	182.5–189.1	5.4033	7.3785	−0.1498	55.42–58.52	
*q18TS-C03–2*	18TS	C03	192.71	191.6–193.5	2.57579	4.4455	−0.1286	56.65–58.52	*cqTS-C03–3*
*q19TS-C03–3*	19TS	C03	192.71	191.7–195.1	5.80692	8.9532	−0.1237	56.65–58.52	
*q17TS-C05–1*	17TS	C05	106.01	103.4–106.9	3.87345	5.2065	0.121	38.83–39.04	*cqTS-C05–1*
*q17TS-C05–2*	17TS	C05	108.11	108.1–108.2	3.6456	5.1333	0.1187	36.71–44.75	*cqTS-C05–2*
*q17TS-C05–3*	17TS	C05	110.41	110.2–110.4	4.04054	5.9714	0.1283	39.04–44.75	*cqTS-C05–3*
*q17TS-C05–4*	17TS	C05	111.11	111.1–112.4	3.96893	5.3291	0.121	39.04–44.75	*cqTS-C05–4*
*q17TS-C05–5*	17TS	C05	117.81	117.6–117.9	3.63475	5.1915	0.119	39.04–44.75	*cqTS-C05–5*
*q17TS-C05–6*	17TS	C05	120.51	120.3–120.6	4.02969	6.0058	0.1292	39.04–44.75	*cqTS-C05–6*
*q19TS-C09–1*	19TS	C09	13.31	13.3–14.3	3.72589	4.1792	0.084	0–0.41	*cqTS-C09–1*
*q19TS-C09–2*	19TS	C09	23.71	23.5–24.8	5.59209	6.1852	0.0993	3.59–4.04	*cqTS-C09–2*

Indel and SNP mutations were further screened using the genome resequencing data of two parents of the KN DH population that were previously obtained [43]. Twenty candidate genes in the CIs of the QTL for TS were found (Table S5), which included genes involved in the sterol biosynthesis pathway (e.g., *BnSQS1*.*C03*, *BnSTE1*.*A03*, and *BnPSAT*.*A08*) and gibberellin (GA) synthesis pathway (e.g., *BnGA1*.*A03*, *BnGA2OX8*.*1A08, BnGA2OX8*.*1*.*C03*). After removing the genes that were repeatedly localized at multiple loci for several different sterols, 43 candidate genes were ultimately identified in the CIs of mQTL (Table S6). Notably, many genes, such as *BnCER3*.*A10* and *Bn3BETAHSD/D2*.*C08*, exhibited synergistic effects on sterol and lipid metabolism. The candidate genes within the CIs of mQTL were abundant in sterol synthases. Notably, *FK, SQE2, SQE3, SQP1, SQP2, SMO2*.*1, 3BETAHSD/D1, 3BETAHSD/D1, DWF4*, and *SMT3*, which have more than one homologous gene, were identified.

### Identification of DEGs using time-series transcriptomics

**Figure 3:**
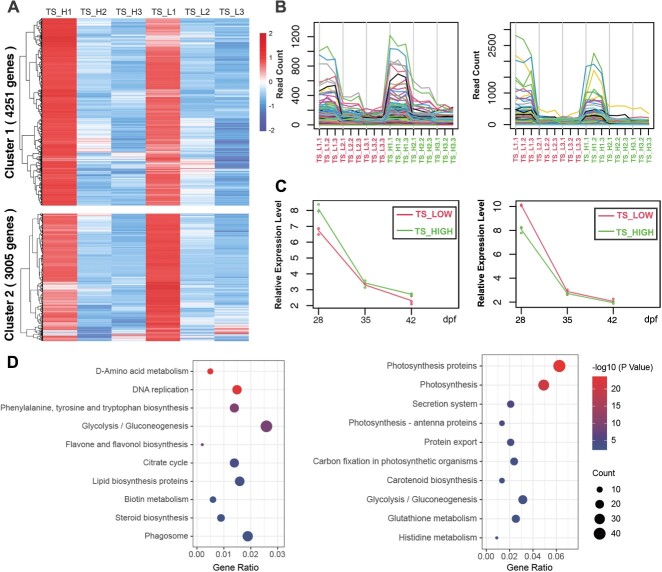
Time-series transcriptomic and KEGG enrichment analysis of Cluster 1 and Cluster 2. (A) Heatmap of relative expression for Cluster 1 (top) and Cluster 2 (bottom), with expression normalized in each row. (B) Read count results for Cluster 1 (left) and Cluster 2 (right). The different lines represent different DEGs. (C) Expression patterns of Cluster 1 (left) and Cluster 2 (right). The green lines indicate high-TS samples, and the red lines indicate low-TS samples. (D) Bubble plot visualization of the KEGG enrichment results for Cluster 1 (left) and Cluster 2 (right). Larger circles denote a greater number of DEGs, with the size of the circles corresponding to the significance of the p value ranging from larger for less significant to smaller for more significant results.

Seeds harvested from a mixed pool of high and low TS in the KN DH population were selected for transcriptomic analysis. To analyze the gene expression patterns during the sterol accumulation period in rapeseed and the differentially expressed genes (DEGs) between high and low sterol content rapeseed, two mixed pools with high and low TS in the KN DH population were selected. Seeds were harvested at 28, 35, and 42 days post-flowering (dpf) and exhibited rapid sterol content accumulation for transcriptomic analysis. Analysis revealed that the 6 groups of 18 RNA-seq results were of good quality, with significant differences between groups and good repeatability within groups (Fig. S5, Table S7). A time-series analysis revealed that 7548 DEGs were identified (Table S8). These DEGs were enriched in pathways related to energy metabolism during seed development, such as photosynthesis and glycolysis, as well as in pathways related to substance metabolism, such as histidine metabolism and the citrate cycle (Fig. S6). Additionally, the time-series analysis grouped the DEGs into a total of nine clusters (Fig. 3A–C; Fig. S7). Regarding the change in sterol content from 20 dpf to seed maturity, the rate of sterol accumulation gradually decreased, reaching its peak (Fig. S8). Cluster 1 (4051 genes with 1988 significant DEGs) and Cluster 2 (3005 genes with 1404 significant DEGs) are considered the most likely to encompass the target DEGs due to their expression trends, which resemble the TS accumulation patterns (Fig. 3C, Fig. S7). KEGG enrichment analysis of DEGs in Cluster 1 and Cluster 2 revealed that genes involved in D-amino acid metabolism; DNA replication; phenylalanine, tyrosine and tryptophan biosynthesis; glycolysis; the citrate cycle; biotin metabolism; lipid biosynthesis; and steroid biosynthesis were enriched (Fig. 3D). The KEGG enrichment pathways in Cluster 2 were significantly different from those in Cluster 1; genes involved in photosynthesis-related pathways, carbon fixation in photosynthetic organisms, carotenoid biosynthesis and glutathione metabolism were enriched. Additionally, among the DEGs, 20 genes were related to lipid biosynthesis (Table S9). These include genes that may belong to the fatty acid desaturase (FAD) family, such as *BnFAD3*.*C03*, *BnFAD3*.*C04*, and *BnFAD7*.*A05*, as well as genes that may belong to the KAS (3-ketoacyl-acyl carrier protein synthase) family, such as *BnKAS1*.*A06* and *BnKAS2*.*C06*.

To investigate the candidate genes and to determine the mechanism underlying the variation in sterol content in rapeseed, the sterol-related genes that could be annotated in all clusters were further analyzed. In total, 108 sterol-related DEGs that might cause differences in TS were identified (Table S10). Combined with the identified candidate DEGs and those genes identified based on the CIs of the TS QTL and mQTL (Fig. S9), several genes involved in sterol biosynthesis were obtained, for example, *Bn3BETAHSD/D1*. *A10*, which was in the CIs of the mQTL (*qST21-A10-1*) and TS QTL (*cqTS-A08-2* and *cqTS-C03-2*). Among the DEGs, *BnSQS1*.*C03* was located in a hotspot region on chromosome C03, which was in the CIs of the consensus QTL produced by the multiple-year TS QTL (*cqTS-C03-2*) as well as multiple mQTL (for example, *qST10-C03-2*, *qST11-C03-1*, *qST12-C03-3*, and *qST15-C03-2*). Additionally, 20 DEGs related to lipid synthesis were also identified (Table S9). For example, *BnGPAT9*.A10 is located within the CIs of the mQTL *qST21-10-1* and *BnKAS2*. C06 is located within the CIs of both mQTL *qST11-16-1* and *qST17-16-1*. These results suggested a relationship between lipid metabolism and sterol synthesis in *B. napus*.

By integrating the sterol-related DEGs contained in all nine clusters and the related genes contained in QTL, a potential regulatory model of sterol biosynthesis and metabolism that could account for the differences in TS and individual sterols was constructed (Fig. S10).

### Construction of potential transcriptional regulatory networks

**Figure 4:**
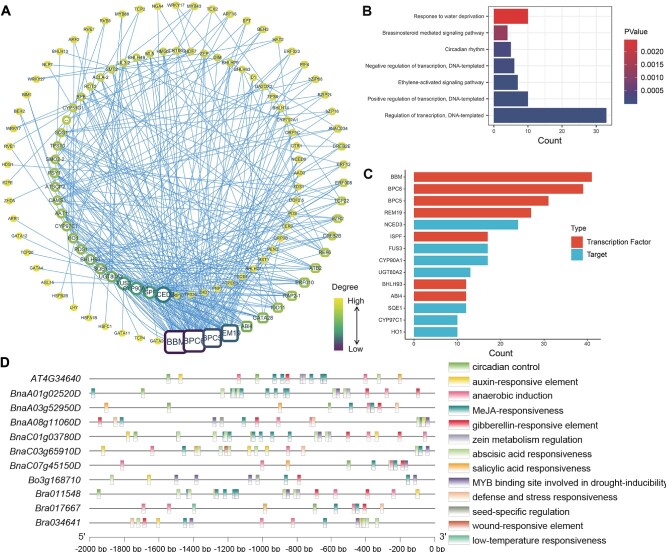
Construction and analysis of transcriptional regulatory networks involving TS-related TFs and target genes and analysis of cis-acting elements in the promoters of *SQS1* homologous genes. (A) Correlation network of TFs and potential target DEGs. The nodes in the inner circle represent the potential target DEGs, and the nodes in the outer circle represent the associated TFs. The size of the nodes correlate with their degree values. (B) Results of KEGG enrichment analysis of TFs. The horizontal coordinates of the bar graph indicate the number of genes. (C) Histogram of nodes with higher degree values. Red indicates TFs, and blue indicates potential target DEGs. (D) Analysis of cis-acting elements in the promoter regions of the *SQS1* homologs. The promoter sequences from −2000 bp to 0 bp are represented on the horizontal line, and different rectangles indicate various response elements.

There are relatively few reports on the regulation of sterol synthesis by TFs in the literature. To analyze the factors influencing sterol synthesis at the transcriptional regulatory level, existing database resources and RNA-seq results were utilized. Through homologous gene comparison with *A. thaliana*, a total of 323 TFs were identified among the 7549 DEGs (Table S11). The most prevalent among these were the MYB family and MYB-related TFs (39 TFs), followed by TFs containing the bHLH domain (34 TFs). Using coexpression analysis, a network of potential regulatory networks based on sterol-related DEGs and TFs was established (Fig. 4A). Mining of the TFs revealed coexpression associations between 91 different TFs and sterol-related DEGs (Table S12). Among these TFs, some TFs are homologs of key genes involved in embryonic development, such as *BBM* (baby boom), as well as homologs of *BPC5* and *BPC6* from the BPC (basic pentacysteine) family, which play significant roles in the development of nutritional and reproductive organs [44–46]. In addition, *REM19*, which is associated with vernalization, and the bHLH family genes *BHLH93* and *ABI4* may also regulate DEGs associated with increased sterol content. After assessment of KEGG enrichment, some TFs were identified as associated with ethylene and BR signaling pathways or playing a role in circadian rhythms and the water stress response (Fig. 4B, C). Among the sterol content-related DEGs, genes associated with the regulation of terpene backbone synthesis, such as homologs of *NCED3*, *FUS3*, *UGT80A2*, and *CYP97C1*, are prevalent. Additionally, sterol synthesis-related genes, including *BnSQE1*.*C09* and *BnCYP90A1*.*A03*, were also identified.

Additionally, using *BnSQS1.C03* as the main research object, the promoter sequences (0 to −2000 bp region) of homologous genes were studied. BLAST analysis revealed that six homologous genes (*BnaA01g02520D*, *BnaA03g52950D*, *BnaA08g11060D* (*BnSQS1*.*A08*), *BnaC01g03780D*, *BnaC03g65910D* (*BnSQS1*.C03), and *BnaC07g45150D*) in *B. napus* were homologous to *AT4G34640* (*AtSQS1*). Further analysis revealed that one homologous gene (*Bo3g168710*) and three homologous genes (*Bra011548*, *Bra017667*, and *Bra034641*) were present in two related species, *Brassica oleracea* (*B. oleracea*) and *Brassica rapa* (*B. rapa*), respectively. Based on prediction and screening, 214 motifs were identified in these 11 promoter regions, and 19 motifs were identified in the promoter region of *BnSQS1.C03* (Fig. 4D). Most of these motifs are response elements of plant hormones, with the most being associated with methyl jasmonate (MeJA) (86 response elements), followed by abscisic acid (ABA) (34 response elements). The fewest of these motifs were related to GA (14 response elements). Motifs related to plant physiological functions primarily include motifs involved in anaerobic induction (20 motifs), MYB binding sites involved in drought inducibility (13 motifs), and circadian rhythm regulation (9 motifs). Of note, in *BnSQS1.C03*, the number of ABA-related motifs (6 response elements) in C03 was greater than that of MeJA-related motifs (4 response elements), indicating that the target gene is more likely to be regulated by ABA. In addition, TFs related to *BnSQS1.C03* that were identified in the transcriptional regulatory network, such as *bZIP16*, exhibit ABA-responsive functions [47]. Additionally, the H3K27me3 demethylase expressed by *REF6* may affect the ABA content by directly reducing the H3K27me3 levels of *CYP707A1* and *CYP707A3*, thereby influencing the ABA regulatory level [48]. The analysis of motifs in the promoter sequences provides some evidence for the reliability of the transcriptional regulatory network.

### *In vitro* and *in vivo* experimental validation of candidate genes

**Figure 5:**
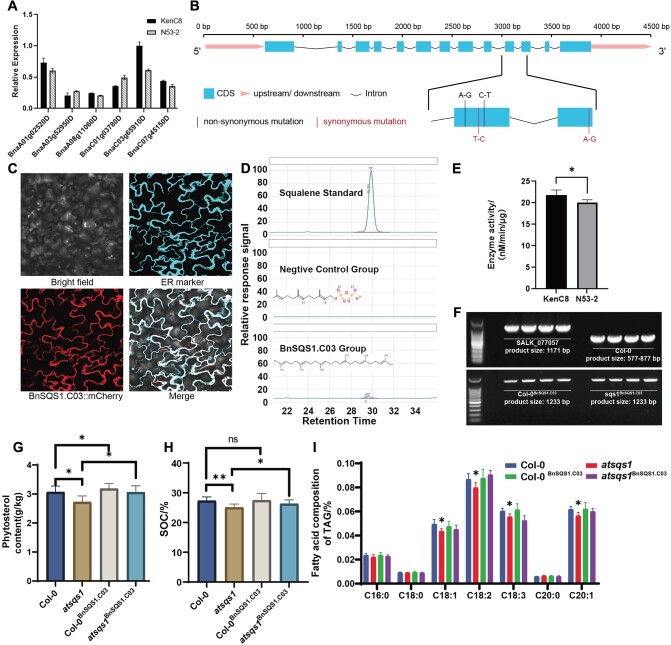
Results of the in vitro and in vivo experimental validation of *BnSQS1*.*C03* activity. (A) Using the rapeseed siliques from the two parents 20 days after flowering as materials, the relative expression levels of the six homologous genes of *SQS1* in rapeseed seeds were detected using qPCR. *ACT7* served as the internal reference. (B) Sequence differences of *BnSQS1.C03* in the gDNA between the two parents. (C) Subcellular localization results. From left to right and top to bottom, images are bright field, ER marker containing CFP tag, *BnSQS1.C03* fused with mCherry tag, and merge, respectively. (D) In vitro enzyme activity experiment results are displayed in the HPLC chromatograph. The y-axis represents the relative response signal intensity, and the x-axis represents the retention time. The three coordinate systems from top to bottom are the squalene standard, the reaction system without SQS, and the reaction result containing *BnSQS1*.*C03* protein, separately. (E) The enzyme activities of proteins synthesized using the two parents as templates. The *y*-axis represents enzyme activity, reported as units of nM/min/μg. (F) The identification results of mutants and overexpression lines. (G) The TS of *A. thaliana* seeds of various lines. The *y*-axis represents sterol content, with units reported as g/kg. Col-0 indicates the wild-type *A. thaliana* Columbia-0, and *atsqs1* represents the tDNA insertion mutant of *AtSQS1*. Col-0^BnSQS1. C03^ and *atsqs1*^BnSQS1. C03^ represent the overexpression lines driven by CaMV35S in Col-0 and *atsqs1*, respectively. (H) The SOC of various *A. thaliana* lines. The y-axis represents SOC, expressed as a percentage. The line designations are the same as above. (I) The fatty acid composition in the TAG of *A. thaliana* seeds from various lines. The *y*-axis represents the content of each different fatty acid, expressed as a percentage. The line designations are the same as above. On the *x*-axis, the groups are categorized based on the different types of fatty acids. Statistical differences were analyzed using *t* tests (^*^P < 0.05, ^**^P < 0.01).

*BnSQS1*.*C03* was selected as a candidate gene after DEG integration analysis based on candidate genes in the CIs of mQTL and TS QTL. Through amino acid sequence alignment, *BnSQS1.C03* showed high amino acid sequence similarity with *Bo3g168710*. Furthermore, after motif analysis, it was found that *BnSQS1.C03* contains all the motifs present in the functionally characterized *AtSQS1* (Fig. S11). The expression of the six homologous genes in the two parents of the QTL mapping population during seed development was examined using 20 dpf siliques as samples, and the results revealed that *BnSQS1*.C03 had exhibited increased expression levels. In addition, a significant difference was noted between the two parents (Fig. 5A). Further comparison of the gDNA sequences of *BnSQS1*.C03 revealed that four SNPs existed in the exons, including two that were missense mutations (Fig. 5B). *BnSQS1*.C03 was localized in the endoplasmic reticulum (Fig. 5C). The protein structures of *AtSQS1* and *BnSQS1*.*C03* predicted by AlphaFold were obtained from UniProt (https://www.uniprot.org/). Alignment revealed that the structures of the two proteins were strongly similar (Fig. S12A). Following methods reported in the literature, the transmembrane regions of *BnSQS1*.C03 were removed for prokaryotic expression analysis, and a protein with a molecular weight of 43 kDa was obtained (Fig. S12B). Subsequent in vitro enzyme activity analysis demonstrated that *BnSQS1*.C03 can catalyze the transformation of farnesyl pyrophosphate (FPP) to squalene (Fig. 5D). Moreover, the activity of the squalene synthase (SQS) enzyme in QT1 (Ken-C8) was greater than that in Q2 (N53–2) (Fig. 5E).

To further validate *BnSQS1*.*C03* activity in vivo, plasmid vectors were designed for transformation into *A. thaliana*, and the T3 seeds of different transgenic lines were obtained (Fig. 5F, Fig. S13, Table S13). The TS in the seeds increased 3.8% (from 3.068 g/kg to 3.185 g/kg) in the T3 lines, and the SOC also increased slightly (0.11%) (from 27.37% to 27.48%). In contrast, TS and SOC decreased by 11.2% (from 3.068 g/kg to 2.724 g/kg) and 8.3% (from 27.37% to 25.09%), respectively, in the homozygous *atsqs1* mutant SALK_077057C (Fig. 5G, H). The contents of seven fatty acids (palmitic acid (C16:0), stearic acid (C18:0), oleic acid (C18:1), linoleic acid (C18:2), α-linolenic acid (C18:3), arachidic acid (C20:0), and ficosenoic acid (C20:1)) were also analyzed. In the *atsqs1* mutant line, the levels of four fatty acids (C18:1, C18:2, C18:3, and C20:1) decreased significantly. However, the levels of these four fatty acids slightly increased in the T3 transgenic lines but did not reach a significant level (Fig. 5I). Complementation experiments were able to effectively restore the wild-type phenotype, demonstrating that *BnSQS1*.C03 could affect the TS content.

## Discussion

### Phytosterol detection methods in rapeseeds and their pharmacological activities

More than 30 components are included in the sterol biosynthesis pathway [49]. However, studies on metabolites, especially secondary metabolites, have been limited by the lack of analytical tools capable of detecting a low content [50]. In the case of *B. napus*, the qualitative and quantitative determination of phytosterols is typically performed using GC–MS. Nonetheless, in the early stages, only a few types of phytosterols, primarily sitosterol, were reported due to their low detection limit [51]. In previous studies on sterols in rapeseed, the focus was on the detection of four sterols with relatively high contents (campesterol, sitosterol, brassicasterol, and avenasterol) and TS, and QTL analysis was performed based on the detection results [31].

The assay and analytical method employed in this study have provided accurate analysis of more than 30 sterols in rapeseed [52]. Twenty-four components, including ursolic acid, were detected in rapeseeds from the KN DH population. Ultimately, 21 components were used for subsequent QTL analysis, of which 17 components were analyzed for QTL in rapeseed populations for the first time. The present results revealed that β-sitosterol, campesterol, and brassicasterol were the most abundant sterols and constituted the major portion of TS, which is consistent with previous reports [53]. Notably, two of these main components show strong biological activity. For example, β-sitosterol has widely reported antioxidant properties as well as anti-inflammatory effects, and it has been extensively developed and applied as a nutraceutical [54]. Moreover, brassicasterol, which is unique to the *Brassica* genus, exhibits anticancer and antiviral effects [55]. In addition to sterols present at high levels, sterols present a lower level also exhibit reported biological activities. For example, ergosterol and its peroxide have been proven to have anti-inflammatory effects and suppress lipid accumulation [56, 57], and sterols can also serve as carriers for drug delivery [58]. Furthermore, fucosterol, which is mainly reported in algae, has received considerable attention given its antidiabetic [59], antioxidant [60], and cytoprotective effects [61]. Therefore, increasing the sterol content in rapeseed has promising development prospects.

### Target gene mining and the potential relationship between TS and SOC based on QTL analysis

The high-density genetic map used in this study was constructed using the KN DH population with SNPs and other types of markers [40, 42], and QTL mapping for several important agronomic traits, including SOC, seed protein content [43], seed glucosinolate content [62], and flowering time [63], was successfully performed. In the present study, using QTL analysis, five consensus QTL on chromosomes A08 and C03 were repeatedly detected over 3 years. These two chromosomes may harbor genetic loci that control multiple sterols, which has also been reported [31]. Additionally, a considerable number of mQTL were also observed to colocalize within these CIs. Due to the larger rapeseed population and higher density map used in this study compared with the previous literature, more mQTL were identified. For example, the QTL for sitosterol, avenasterol, and brassicasterol, which are located on chromosomes C01 and C09, A05 and C01, and C04 and C07, respectively, were first reported in this study. Additionally, TS QTL located on chromosomes C05 and C09 were also reported for the first time in this study.

In previous studies, several sterol biosynthesis-related genes in the major QTL were identified [32]. For example, a homolog of *CYP710A2* involved in catalyzing the desaturation reaction at position C-22 to produce stigmasterol and brassicasterol was identified at a multiphenotypic colocalization QTL on A04 [64]. Additionally, an *SMT2* homolog on chromosome A06 was identified that may catalyze the methyl transfer from S-adenosyl-methionine to the methylene group of 24-methylene lophenol, resulting in the formation of 24-ethylidene lophenol [23]. In this study, benefiting from the good linear relationship between the physical and genetic maps of the KN DH population [43], it became possible to rapidly obtain candidate genes within the CIs of QTL and make functional predictions based on the *A. thaliana* homologs. According to the QTL analysis, two chromosomes, A08 and C03, emerged as crucial genetic loci regulating sterol content. Furthermore, a significant number of the screened genes were obtained, some of which are enzymes involved in the sterol synthesis pathway (including *SQS1*). In plants, squalene is the first committed precursor for the synthesis of essential mevalonic acid-derived isoprenoids, such as sterols, BRs, and triterpenes. SQS is a membrane-bound enzyme that catalyzes the synthesis of squalene from the C-15 allylic compound farnesyl diphosphate (FPP) through a two-step reaction [65]. In this study, homologs of *SQS1* in the hotspots on A08 and C03 were detected (designated *BnSQS1*.*A08* and *BnSQS1*.*C03*). These two genes were also detected in the CIs of several mQTL (*qST10-C03-2*, *qST12-C03-3*, *qST15-C03-2*, *qST16-C03-1*, *qST2-C03-2*, *qST3-C03-1*, *qST4-C03-1*, *qST6-C03-1*, *qST8*-C03-*2*, and *qST9-C03-2*). In *A. thaliana*, *STE1/DWF7* encodes a similar functional protein (Delta(7)-sterol-C5(6)-desaturase 1) as *ERG3* in yeast [66], and its homologous gene *BnSTE1*.*A03* was detected in *cqTS-A03–2*.

Among the genes located in the CIs of the QTL for TS, sterol transport-related genes have also received increased amounts of attention. Oxysterol-binding proteins (OSBPs) and OSBP-related proteins (ORPs) function to transfer newly synthesized sterols from the endoplasmic reticulum to the plasma membrane [67], and 12 putative *ORP* genes have been identified in *A. thaliana* [68]. Furthermore, these proteins are also involved in membrane trafficking [69] and cell cycle progression [70]. In the present study, four *ORP* genes, including *cqTS-A03-1*, *cqTS-C03-2*, and *cqTS-C05-2*, were detected in the CIs of TS QTL. Except for *BnORP3B*.*2*.*C05*, other *ORP* genes were also detected in the CIs of mQTL, suggesting that ORPs might play an important role in the regulation of sterol content in rapeseed.

Given that terpenoids, the precursors of sterols, are also important precursors for GA synthesis [71], these genes related to GA metabolism were also identified as candidate genes. Notably among these is the, *GA2OX8*, which catalyzes the deactivation of C-20-GAs [72]. In *A. thaliana*, mutants deficient in ga2ox8 produce greater levels of active GAs than the wild type [73]. Among the TS QTL, the genes homologous to *BnGA2OX8*.*A08, BnGA2OX8*.*1*.*C03*, and *BnGA2OX8*.*2*.*C03* were also identified in *B. napus*. In addition, *BnGA1*.*A03*, a homolog of *GA1* (a key enzyme for GA synthesis), was also found in the CI of *cqTS-A03–2*. It was thought that there was a signal crossover between BRs and GA at the transcriptional level [74, 75]. However, few reports on the interaction between GA biosynthesis and sterol biosynthesis exist. The present results suggest the possibility of a mutual influential relationship between sterols and GA from the perspective of secondary metabolite flow.

Based on correlation analysis, no significant relationship between TS and SOC was found. In contrast to TS, SOC was significantly positively correlated (*P* < 0.01) with four compounds (cycloartenol, squalene, 24-methylenecycloartenol, and cycloeucalenol). A considerable number of mQTL that were colocalized with QTL for SOC were identified on A08 and C03. In previous studies, a large number of SOC-related QTL were also localized on A08 and C03 [76], some of which were colocalized with the TS QTL and mQTL obtained in this study [43]. A similar study of the correlation between SOC and sterol components in sunflower seeds in 2012 also revealed colocalization of QTL for cycloeucalenol and squalene with SOC [28]. These QTL also included genes involved in acyl-lipid metabolism in *B. napus* and *A. thaliana*. The candidate genes *HMG1* and *ACC1* are coregulated by *AMPK* [25]. There are also reports that the overexpression of certain fatty acid synthesis-related genes leads to the upregulation of the expression of genes involved in sterol biosynthesis [26]. Alterations in the membrane sterol composition, but not deficiencies in BRs or sterol esters, could compromise LD accumulation in leaves and seeds [27]. In Arabidopsis, the *cpi1-1* (cyclopropyl isomerase 1) mutant displayed elevated expression of both auxin-responsive and biosynthesis genes [77]. This phenotype was recapitulated upon treatment with cycloeucalenol, a precursor of CPI1. Considering the studies mentioned above, it could be hypothesized that lipid accumulation in rapeseed is strongly correlated with the overall content and proportion of sterols.

### Regulatory networks of sterol biosynthesis were successfully constructed using multiomics analysis

Using a combination of QTL and transcriptome analysis has demonstrated to be a more efficient approach for identifying candidate target genes associated with complex traits [78]. DEGs that are correlated with changes in phenotype can be rapidly screened using a time-series transcriptome strategy [79]. In this study, the genes involved in the steroid biosynthesis pathway were notably included in the findings. Moreover, pathways involved in lipid biosynthesis, DNA replication, amino acid anabolic processes, and glycolysis were also identified. The DNA replication pathways as well as amino acid metabolism-related pathways identified suggest that the sample collection period coincided with the fastest growth period of the seeds [80]. Similarly, many photosynthesis-related pathways and lipid biosynthesis processes that were enriched during this period were due to the requirements for carbon sources and lipid synthesis [81]. Unexpectedly, enrichment of the glycolytic pathway was observed, and it has been reported that in the *wrinkled1* mutant of *A. thaliana*, disruption of the glycolytic pathway eventually affects SOC [82]. In *B. napus*, glycolysis provides 90% of the plastid acetyl-CoA precursors for seed embryo development [83]. Additionally, it is important to note that a portion of sterol precursors (known as terpenoids) synthesized through the MEP pathway rely on the glycolytic pathway [84].

All DEGs related to the sterol biosynthesis pathway, terpenoid backbone biosynthesis pathway, and BR biosynthesis pathway were collected, and these DEGs were integrated with the candidate genes identified in the CIs of TS QTL and mQTL to model the regulatory network. Most of the overlapping key genes are involved in the sterol biosynthesis pathway, such as B*nSQS1*.*C03*, *Bn3BETAHSD/D1*.*A10*, and *BnSMO2-1*.*A10*. The homolog of *Bn3BETAHSD/D1*.A*10* in *A. thaliana*, *3BETAHSD/D1*, encodes beta-hydroxysteroid dehydrogenase [85]. This gene potentially regulated lipid *de novo* bulk synthesis in the endoplasmic reticulum. *SMO2-1* encodes methylsterol monooxygenase 2-1, which is involved in sterol biosynthesis by catalyzing the removal of the second methyl group at the C-4 position [86]. The *BnSMO2-1*.*A10* is a homolog of *SMO2-1* on chromosome A10, and embryonic lethality was detected in *A. thaliana* with double *SMO2–1* and *SMO-2* mutations [87]. In addition to genes involved in the sterol biosynthesis pathway, *BnHDS*.*A10*, which is involved in the terpenoid backbone biosynthesis pathway, was also identified as involved in the MEP pathway to produce famesyl-PP [88]. A homolog of *BnCPD*.*A03*, a *CPD* gene, is a key gene for BR synthesis; it catalyzes C-3 oxidation, and defects (such as cell elongation) can occur in *A. thaliana* mutants [89].

The synthetic pathways of phytosterols have been relatively well studied, but reports on their transcriptional regulation are lacking. In Arabidopsis, studies have demonstrated that the NAC TF jungbrunnen 1 (*JUB1*) and the bHLH TF phytochrome interacting factor 4 (*PIF4*) are capable of modulating the concentrations of free sterols and brassinolide through direct transcriptional regulation of *DWF4* [90, 91]. Moreover, the MYB TF *GmMYB14* has been shown to influence gene expression by binding to the AC element located upstream of *GmBEN1*, thereby impacting brassinolide levels [92]. In this study, for the first time, a potential regulatory network was constructed based on sterol-related DEGs and TFs. Through the analysis of the nodes, the TFs that are likely to regulate multiple sterol synthesis-related genes were identified. One such TF is *BBM*, which encodes an AP2 domain TF. In *B. napus*, two copies of *BBM* were initially identified. Early studies of *BBM* demonstrated its ability to activate signal transduction pathways that induce embryonic development in differentiated somatic cells [93, 94]. Another important TF is basic pentacysteine (*BPC*), which is believed to play a role in various plant growth and developmental processes. In *A. thaliana*, mutants with multiple BPC alleles exhibit diverse developmental defects, such as dwarfism, small rosettes, early flowering, aberrant ovules, unopened floral buds, and high sterility [95–97]. Previous studies have demonstrated that disruption of multiple BPCs results in altered cytokinin and ethylene sensitivity [95, 97, 98], and *BPC6* of *A. thaliana* was also shown to target the promoters of all major BR signaling components [99]. Furthermore, their coexpression relationships with multiple TFs potentially indicate that target functional genes are regulated by various hormonal or stress responses during seed development. Based on network analysis, it could be concluded that terpenoids, precursors of sterols, might be more susceptible to regulation by TFs. In the sterol synthesis pathway, *BnSQE1*.*C09* and *BnCYP90A1*.*A03* are influenced by more TFs. These findings provide additional avenues for further research on the regulation of sterol content and the enhancement of sterol biosynthesis. Notably, after analyzing the cis-acting elements in the promoters of *BnSQS1.C03* and its homologous genes, it becomes more apparent that this target gene may be influenced by a variety of plant hormones and regulated by a multitude of TFs. Multiple ABA transcription regulatory elements are present in the promoter region of *BnSQS1.C03*, suggesting a significant regulatory role for ABA. Previous studies have generally shown that ABA and BRs have antagonistic effects on each other, with BRs acting at an earlier stage in the signaling pathway [100–102]. However, in this study, analysis of transcriptional regulatory networks and promoter sequences revealed that *SQS1*, a key gene in BR biosynthesis, is coexpressed with several ABA-related TFs and possesses multiple ABA-responsive elements in its promoter regions. This result suggested that there may be a more complex interregulatory pattern involving biosynthesis and metabolism between ABA and BRs.

### *BnSQS1.C03* simultaneously affects TS and SOC in rapeseeds

The previous literature on *SQS1* includes studies on its enzymatic function and a limited number of studies related to its physiological role in plants. *SQS1* expression increases in response to salt stress at different concentrations and is stimulated by arbuscular mycorrhizal symbiosis [103, 104]. Based on phylogenetic tree analysis, the relationships between the *SQS1* homologous genes in *B. napus* and those in two closely related species (*B. oleracea* and *B. rapa*) were investigated. Among them, *BnSQS1*.C03 and *BnSQS1*.A08, which have received considerable attention in the past, both show stronger similarity to *Bo3g168710*, suggesting that these two genes may have been acquired from *B. oleracea*. *In vitro* experiments demonstrated that *BnSQS1*.*C03* plays a crucial role in the synthesis of squalene, a key intermediate in the sterol biosynthesis pathway [105]. These results suggest that the differences in protein activity produced from the cDNA templates of the two parents may partially explain the differences in TS levels between the two parental lines. In *in vivo* experiments, the increased expression of *BnSQS1*.*C03* in *A. thaliana* seeds led to a significant increase in TS, which consequently resulted in a modest increase in SOC. The abrupt decrease in sterols in the seeds of the *atsqs1* mutant led to a significant reduction in SOC, and *BnSQS1*.*C03* overexpression reversed this phenomenon completely. Complementation experiments in mutants further confirmed the similar functions of *BnSQS1*.*C03* and *AtSQS1*. Although the trait investigation in the population did not show a significant statistical correlation between TS and SOC, the *in vivo* validation experiments validated not only that *BnSQS1*.*C03* is a pivotal gene in sterol biosynthesis but that it also has a significant association with SOC in rapeseed. Although there are literature reports of sterol and fatty acid synthesis-related genes showing similar expression trends under the same treatments [106, 107], this study is the first to observe changes in fatty acids by directly altering the TS in plants. This finding suggests that there might be a complex regulatory network between the sterol and SOC in plants and that *BnSQS1*.*C03* could represent a valuable target for further understanding this connection.

## 1. Conclusion

Increasing the phytosterol content is of great importance in rapeseed breeding at present. Analysis of the genetic mechanism of phytosterol synthesis would be helpful for further breeding in rapeseed. Comprehensive QTL mapping was conducted on the TS and 21 sterol-related components. In total, 24 TS QTL and 157 mQTL were identified, with multiple stable and pleiotropic loci detected on A08 and C03, which are considered hotspot regions. Using an integrative multiomics approach, *BnSQS1.C03* emerged as a pivotal gene with the potential to simultaneously influence the sterol and oil contents in seeds. These results provide a solid basis for the future cultivation of rapeseed with higher contents of phytosterols.

## Materials and methods

### Plant materials and growing condition

The KN DH population used in this study was a DH population derived from the microspore culture of F1 plants from the hybridzation of Ken-C8 and N53-2 [42], and the genetic linkage map with 3106 SNP bins (encompassing 17 978 SNPs and 101 non-SNP markers (SSR and STS)) [43] was used for QTL analysis for TS and all the individual sterol content. The field experiments adhered to the normal agricultural practice. The seeds that harvested in the year from 2017 to 2019 were used for TS content measurement, and the seeds in 2019 were also used for individual sterol content analysis.

### Measurement of the sterols and oil content in seeds

The protocol for sterol extraction and measurement is according to the method of Xu *et al.* [52] with minor modifications. Ground seeds of different KN DH lines were placed in test tubes, and the cholesterol internal standard was added. The seeds were first saponified with an KOH ethanol solution, after which the hexane and ddH_2_O were added. Supernatants from the three extractions were collected in tubes and dried using the nitrogen blowing concentrator. Then the derivatizer MSHFBA:1-MIM (95:5, v/v) was added to the tubem, and the mixture was derivatized at 75°C. The sample was ultimately dissolved in hexane and then analyzed using a DB-5MS (30 m × 0.25 mm × 0.25 μm) column in gas chromatography–mass spectrometry (GC–MS). Measurement data was acquired using a combining the selected-ion monitoring and scanning mode. The qualitative analysis of sterols was performed by standard quality spectrograms and retention times, and the internal standard method was used for quantification. Quantitative and qualitative ions were referenced according to the method of Xu *et al*. [52].

The Liebermann–Burchard reaction was employed for TS analysis. Extraction and separation of TS were conducted according to the protocol described in the previous GC–MS section. After drying of the extract, the resulting residue was subsequently dissolved in methanol. Acetic anhydride was added into the solution and mixed up. Following the addition of concentrated sulfuric acid to the mixture and slight shaking, the absorbance was detected under 530 nm immediately after the solution was cooled to room temperature. β-sitosterol was used as the control substance for quantitative analysis.

The SOC and fatty acid compositions of KN DH population that used for correlation analysis with 21 individual sterols and TS in 3 years were from the Chao et al. [43]. Correlation heat maps were generated using the corrplot package in R software.

### QTL analyses and candidate gene identification

QTL analysis for each trait were performed using a composite interval mapping (CIM) with Windows QTL Cartographer 2.5 software [108]. The scan walking speed was 1 cM and the window size was 10 cM. The LOD threshold for significant QTL was determined by a 1000-permutation test based on a 5% experiment-wise error rate. All detected QTL were denoted as significant identified QTL. QTL integration was carried out using meta-analysis with BioMercator 4.1 software [109]. The identified QTL were named by combining the phenotype and the chromosome number, e.g., *qST14-A04-2*. Consensus QTL were designated by the initial letters “cq,” such as *cqTS-C03-2*, representing the second consensus QTL identified for TS on chromosome C03.

The alignment of the genetic map to the physical map, and the candidate gene identification followed the same methods described by Chao *et al*. [44]. Utilizing the collinearity of the high-density genetic map and “Darmor-bzh” reference genome (http://www.genoscope.cns.fr/brassicanapus/data/), the genome regions corresponding to the CIs of QTL were identified by using closely linked SNP within the CIs of QTL [110]. Candidate genes within CIs of QTL were regarded as candidate genes. These genes were annotated and named based on the homologous gene of *A. thaliana* and their respective chromosome locations (e.g., *BnFK*.*A04* for the ortholog of *AtFK* on ChrA04). The orthologous of candidate genes and their annotation were obtained by BLASTn based on *A. thaliana* database (http://www.arabidopsis.org/).

Sterol content-related genes were collected from Gene Ontology database (http://geneontology.org/) by searching the keywords such as “sterol biosynthetic process”, “sterol metabolic process”, “sterol transport process”, and “terpenoid biosynthesis”. Candidate genes in the CIs of QTL were screened using the collected sterol-related genes to identify potentially important target genes within the intervals. Visualization of QTL intervals and candidate genes was conducted using Circos software [111].

### Gene expression analysis of seeds during different developmental stages

Thirty lines with relatively higher TS and 30 lines with relatively lower TS in KN DH population were selected, respectively. Seeds from these sixty lines at 28 dpf, 35 dpf, and 42 dpf were collected and stored at −80°C for further transcriptomic analysis. Three replications were conducted for all the materials, resulting in a total of 18 samples that were sent for RNA sequencing in Qiantang Biotech Corporation (Suzhou, Jiangsu Province, China).

The original data were processed to remove the reads of low quality and joint contaminations to ensure the reliability of the results. HISAT was used to align clean reads to the reference genome (ZS11) [112]. Bowtie2 was utilized for aligning clean reads to the reference sequence, followed by RSEM for calculating the expression levels of genes and transcripts. According to the requirements, maSigPro [113] and DEseq2 [114] algorithms were applied to detect the time-series DEGs.

Potential TFs of sterol-related DEGs were identified using the TF regulation network module of the BnIR (http://yanglab.hzau.edu.cn/BnIR) website. Cytoscape was employed to construct the network of TFs and their functional target genes. The degree value of each node, representing the number of associations with other nodes, was obtained from Cytoscape. Enrichment analysis and visualization of KEGG and GO were performed using clusterProfiler and ggplot2 in R software [115]. A regulatory map was constructed based on the pathway of *A. thaliana* in the KEGG pathway database (https://www.genome.jp/kegg/pathway.html).

### cDNA cloning and sequence alignment

In the present study, the coding sequence (CDS) region of the candidate gene *BnSQS1*.C03 was cloned from cDNA in two parents (KenC8 and N53–2) of mapping population. The CDS of *BnSQS1*.C03 was cloned using the PMD18-T vector and *Escherichia coli* (*E. coli*) DH5α. Homologous sequences were aligned using the BLAST function of the online Brassica database (http://brassicadb.cn/#/BLAST/). Nucleotide sequence translation, alignment, and amino acid sequence alignment were performed using MEGA 7. Gene structure was analyzed and mapped using the gene structure display server (GSDS), an online software program (http://gsds.gao-lab.org/). Promoter cis-acting element analysis and amino acid sequence motif analysis were performed using the corresponding prediction tools from the MEME (https://meme-suite.org/meme/tools/meme) and PlantCARE (https://bioinformatics.psb.ugent.be/webtools /plantcare/html/). The sequences of all primers used in the study are listed in Table S14.

### Expression of soluble recombinant forms of *BnSQS1*.*C03* in *E. coli*

To express *BnSQS1*.*C03* in *E. coli*, the following steps were performed: The CDS of *BnSQS1*.*C03* was inserted into the pET28a vector, and the recombinant plasmid was then transformed into BL21(DE3) *E. coli* cells. The transformed cells were cultured in 2 × YT medium (containing 16 g/L yeast extract, 16 g/L tryptone, 5 g/L sodium chloride) with kanamycin. Induction was initiated with isopropyl β-D-1-thiogalactopyranoside (IPTG) at 16°C. After centrifugation and resuspension in buffer A (containing 0.27 g/L potassium dihydrogen phosphate, 1.42 g/L disodium hydrogen phosphate, 8 g/L sodium chloride, 0.2 g/L potassium chloride, and adjusted to pH 7.4 with concentrated hydrochloric acid). Cell lysis was achieved by sonication. The insoluble fraction was discarded, and the supernatant was purified using Ni-agarose resin, followed by elution with imidazole gradients. The purified proteins were analyzed by SDS-PAGE and concentrated using an ultrafiltration centrifuge.

### Assay for SQS activity

The SQS activity assay was performed as described with minor modifications [65]. The reaction mixture, totally 100 μL, comprised of 25 μM FPP, 2 mM NADPH, 10 mM dithiothreitol, 5 mM MgCl_2_, 15 μg squalene synthase (SQS), and 0.1 M PBS buffer (containing 0.027 g/L potassium dihydrogen phosphate, 0.142 g/L disodium hydrogen phosphate, 0.8 g/L sodium chloride, 0.02 g/L potassium chloride). The reaction was incubated for 30 minutes at 30°C and then terminated by boiling for 5 minutes. The sample was extracted with *n*-hexane, concentrated, and dissolved in acetonitrile. Detection was performed using an Agilent 1200 LC with a VWD detector, on a ZORBAX Eclipse C18 column, using 100% acetonitrile as the mobile phase at room temperature, with a detection wavelength of 210 nm.

### Subcellular localization experiment

The CDS of *BnSQS1*.C03 was cloned into the pCAMBIA-1303 plasmid, which contains the red fluorescent protein (mCherry) gene at the C-terminus, and the expression is driven by the *Cauliflower Mosaic* Virus (CaMV) 35S promoter. The subcellular localization vector was transformed into the *Agrobacterium tumefaciens* strain GV3101, and then it was infiltrated into the leaves of *Nicotiana benthamiana* following the previously reported procedure [116]. The fluorescent signal was detected using an FV1000 laser scanning confocal microscope (Olympus, Japan).

### Vector construction and transformation into Arabidopsis

The wild-type *A. thaliana* that used in the experiment was Columbia-0 (Col-0). The T-DNA insertion mutant SALK_077057C of the homologous gene *AtSQS1* in *A. thaliana* was purchased from the Arashare website (http://www.arashare.cn). The *BnSQS1*.*C03* gene was integrated into the pCAMBIA-1303 vector through homologous recombination, with gene expression driven by the CaMV35S promoter. The recombinant vector was transformed into *A. tumefaciens* strain GV3101 by electroporation. The flower-dip method was used to transform the gene into Columbia-0 and SALK_077057C. Homozygous T2 and T3 generation transgenic lines were used for subsequent analysis.

## Supplementary Material

Web_Material_uhae196

## Data Availability

The data that support the findings of this study are accessible in the sequence read archive (SRA) of the National Center for Biotechnology Information (NCBI) (https://www.ncbi.nlm.nih.gov) with the accession number PRJNA1078081.
