# Supplementary material for: Integrating QTL mapping and transcriptomics to decipher the genetic architecture of sterol metabolism in Brassica napus L
Source: Hortic Res. 2024 Jul 24;11(9):uhae196. doi: 10.1093/hr/uhae196 (PMC11384122; doi:10.1093/hr/uhae196)
Supplement: Web_Material_uhae196 [file web_material_uhae196.zip › Supplementary figures.docx]

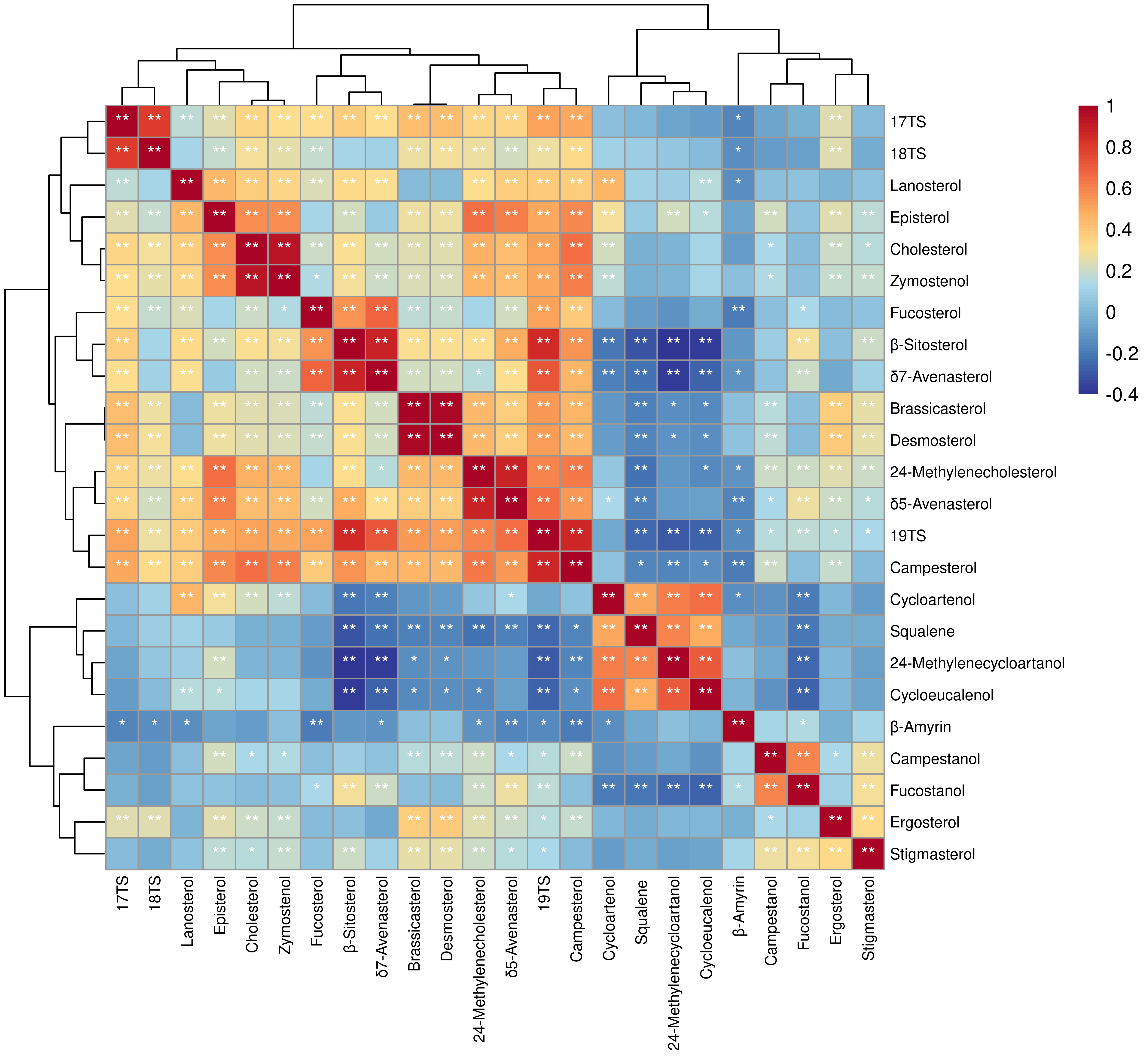


**Figure S1** The correlation coefficient is presented using color, with red indicating a positive correlation, blue indicating a negative correlation, and light blue indicating a slight positive correlation. The darker the color, the stronger the correlation. The results of the cluster analysis are presented using branches at the edges of the heat map. (Levels of significance are as follows: *p < 0.05, **p < 0.01, and ***p < 0.001.)


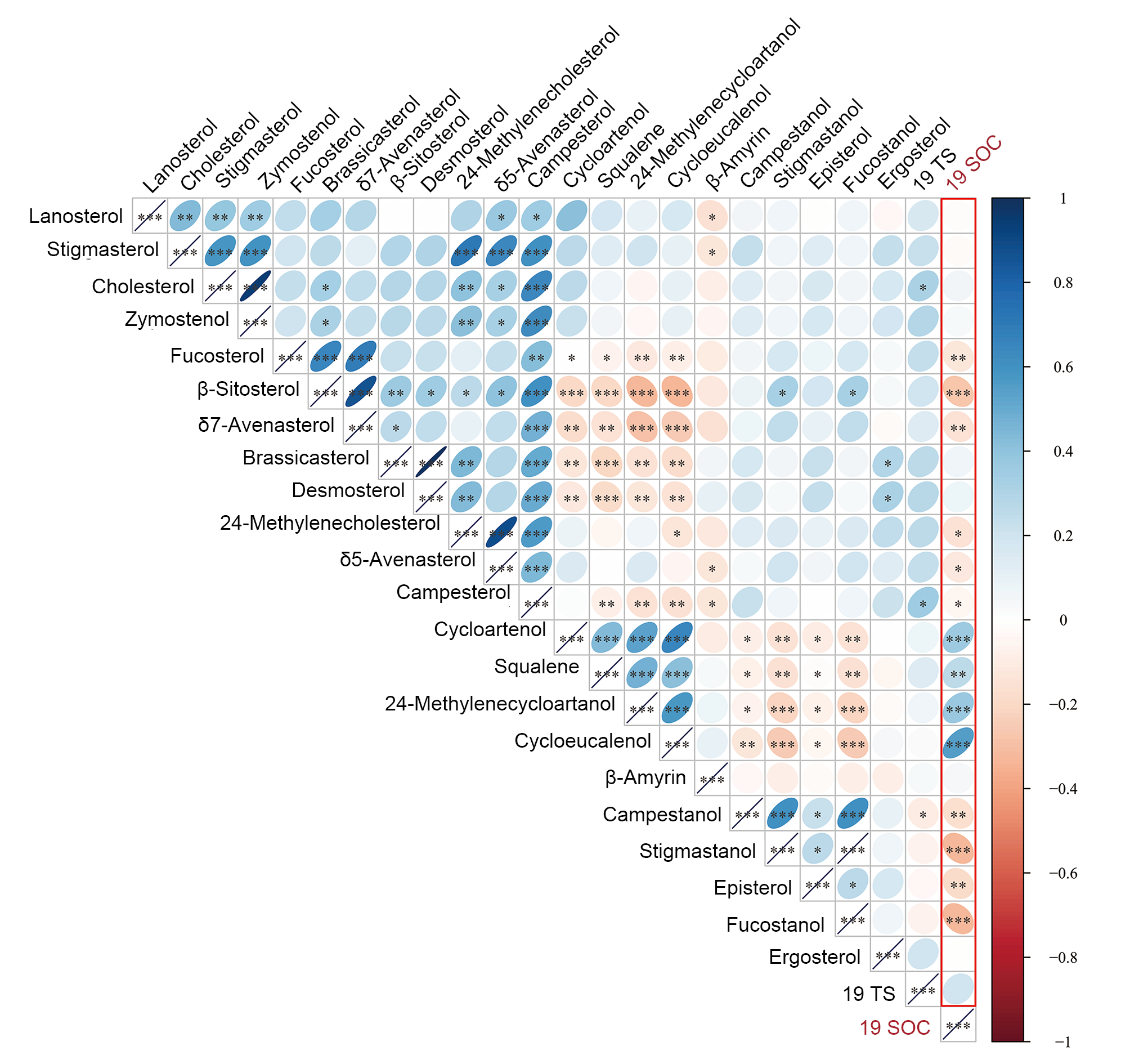


**Figure S2.** Correlation analysis between sterols and SOC. Correlations were visualized with ovals. Darker

ovals indicate stronger correlation, blue is positive correlation, red is negative correlation, and narrower ovals

indicate stronger significance. (Levels of significance are as follows: *p < 0.05, **p < 0.01, and ***p < 0.001.)


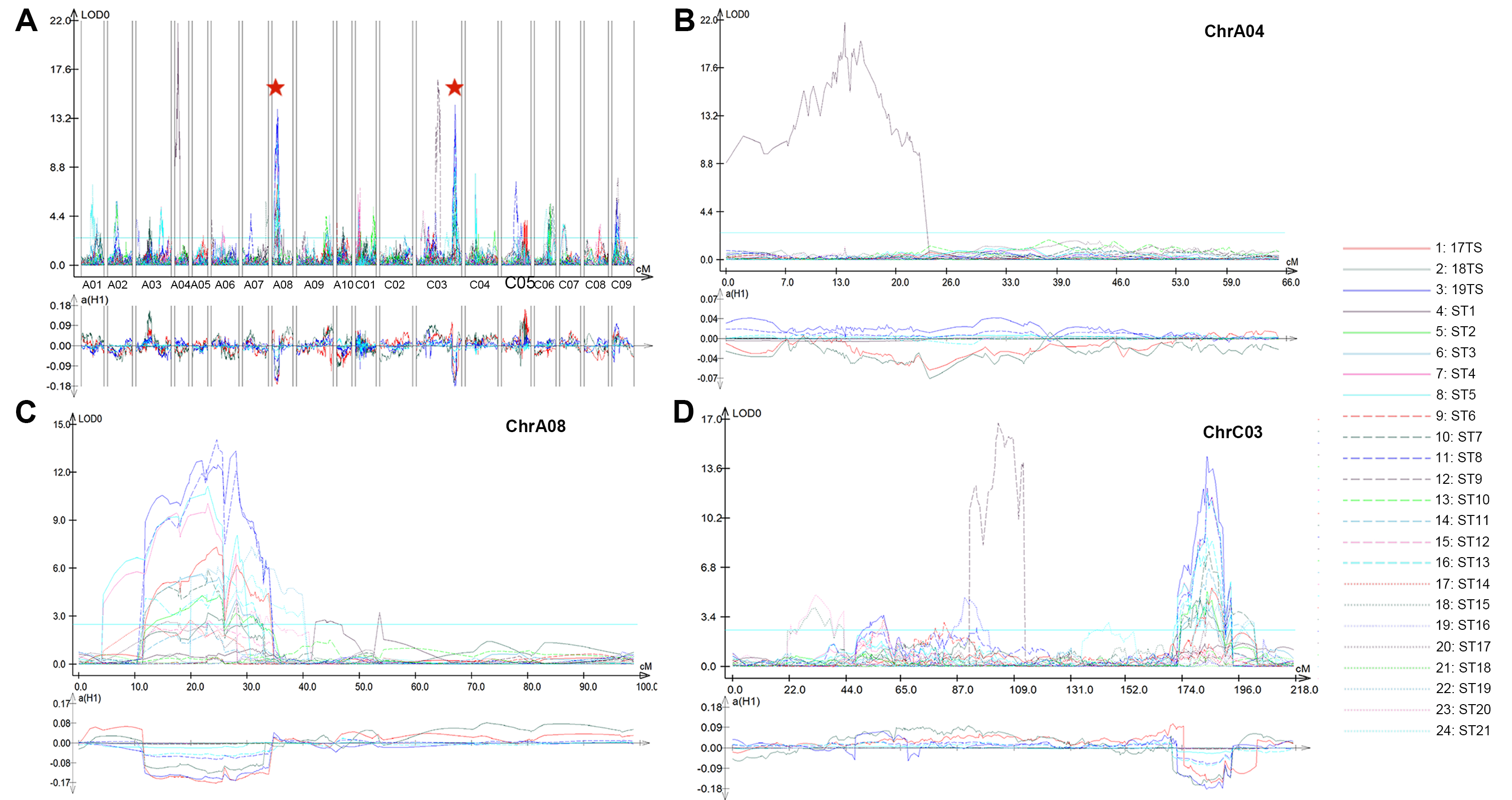


**Figure S3**. The QTL mapping results, including TS obtained in three years and 21 individual sterols. The

different colored lines in the figure represent different traits. (A) Mapping results for all traits on 19

chromosomes. On the upper coordinate axis, the vertical axis represents LOD values, while the horizontal axis

represents genetic length of the 19 chromosomes. On the lower coordinate axis, the vertical axis represents

additive values, while the horizontal axis still represents genetic distance. In the figure, red pentagrams indicate the regions where the hotspot regions are located. (B-D) Mapping results for all traits

on chromosomes A04, A08, and C03. The vertical axis of the coordinate axis represents LOD values or

additive values, while the horizontal axis represents the genetic distance on each chromosome.


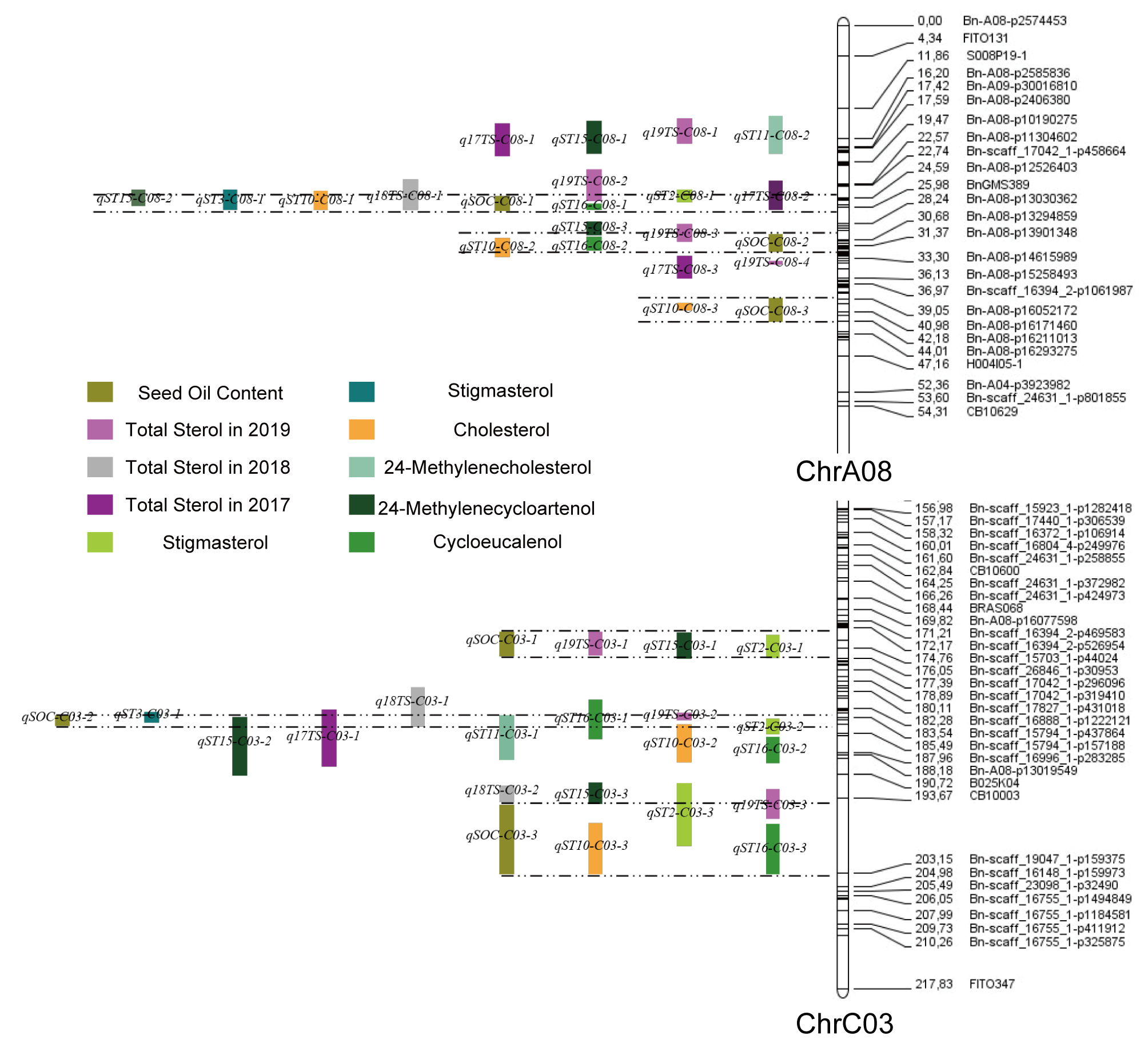


**Figure S4.** Sterol QTL results were compared with SOC results. Vertical bars represent chromosomes, and the lines on chromosomes represent markers near QTL. The color bars on the right side of the chromosomes represent QTL detected from different traits.


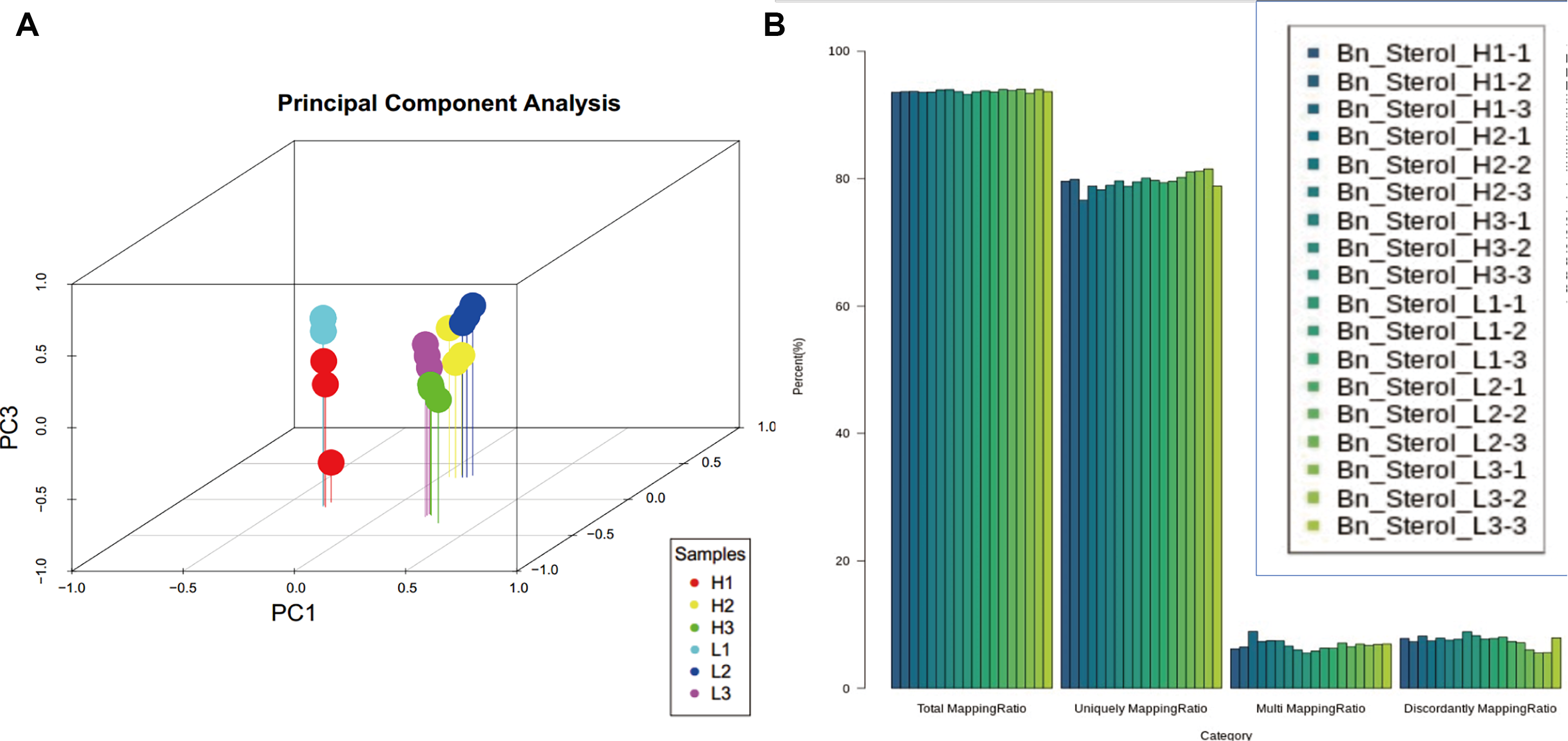


**Figure S5.** RNA-seq sequencing quality analysis. (A) Principal Component Analysis (PCA) of the samples. (B) Mapping ratio of the RNA-seq samples.


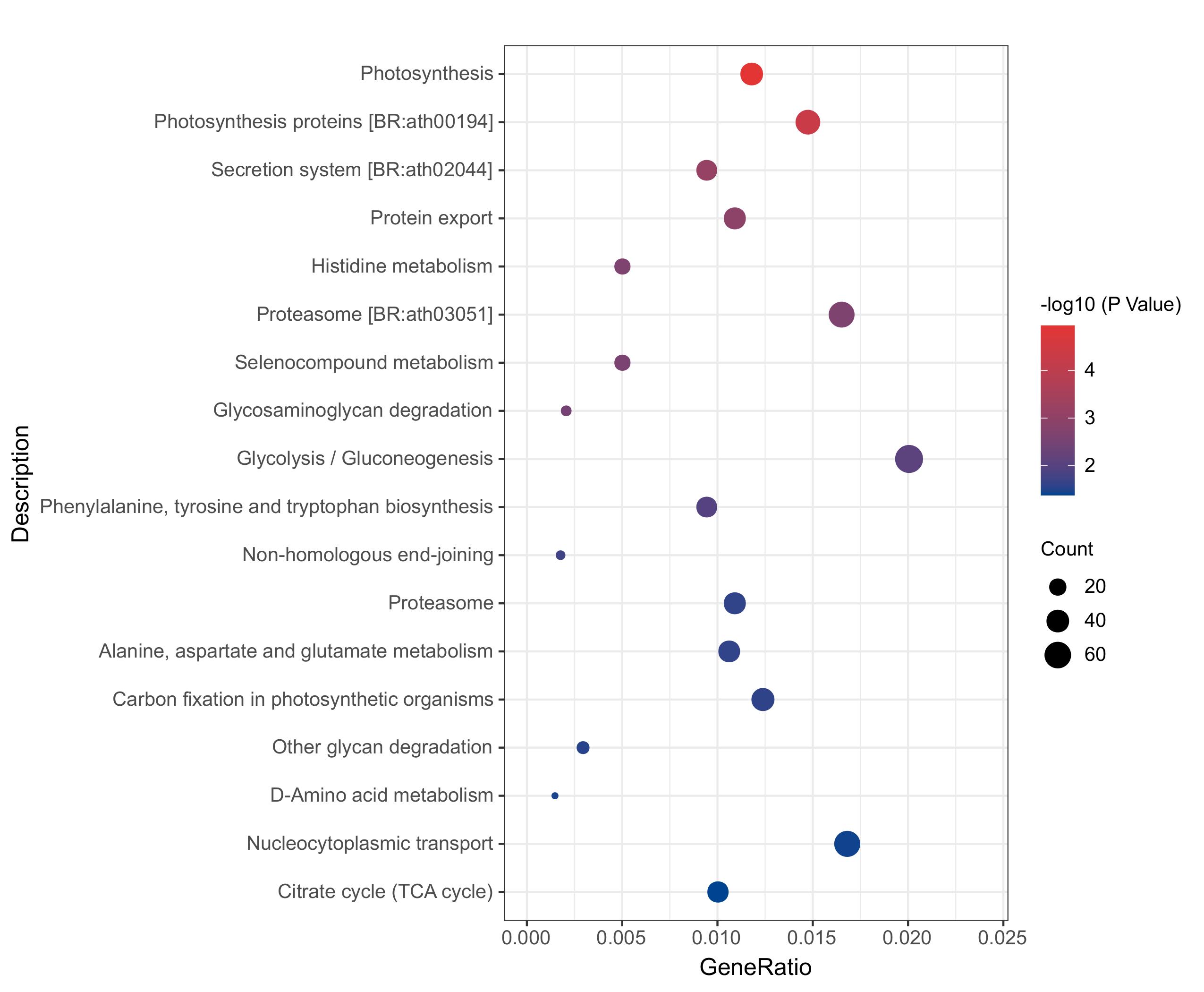


**Figure S6.** The KEGG enrichment of all DEGs. Bubble plot visualization of the KEGG enrichment results. The larger circles indicate that the corresponding terms have more DEGs. The color of the circles ranges from blue to red, indicating that the corresponding terms have a relatively lower P value, which means they are more significant.


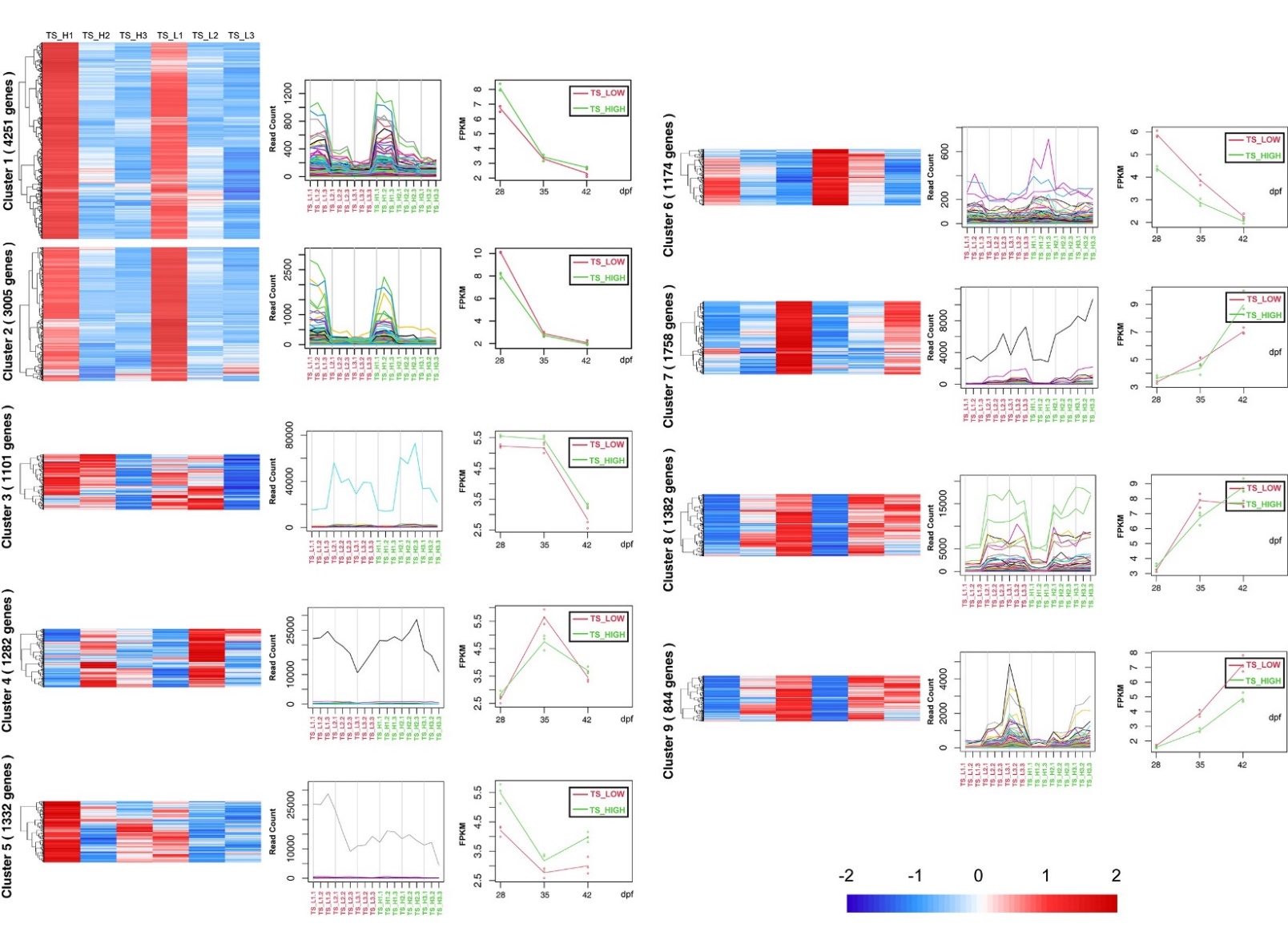


**Figure S7.** The results of time-series transcriptome analysis for nine clusters. The figures are divided into nine

groups, with the number of genes in each cluster labeled on the far left. The heat map represents the relative

expression levels of each group, normalized by rows. The line plot in the middle represents the read count of

genes within each cluster, with different colors indicating different lines. The line plot on the right represents

the expression pattern of this cluster, with the green line indicating the high TS group and the red line

indicating the low TS group.


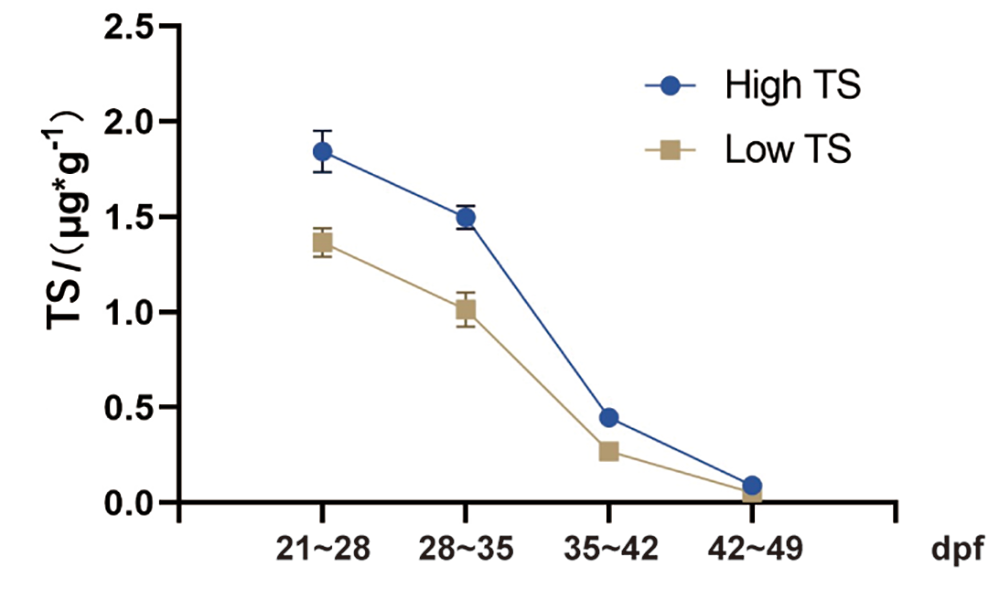


**Figure S8.** The accumulation rate of TS in rapeseed silique from 21 to 49 days after flowering. The y axis represents the increase in TS levels within the time interval, with the unit being μg/g (micrograms per gram).


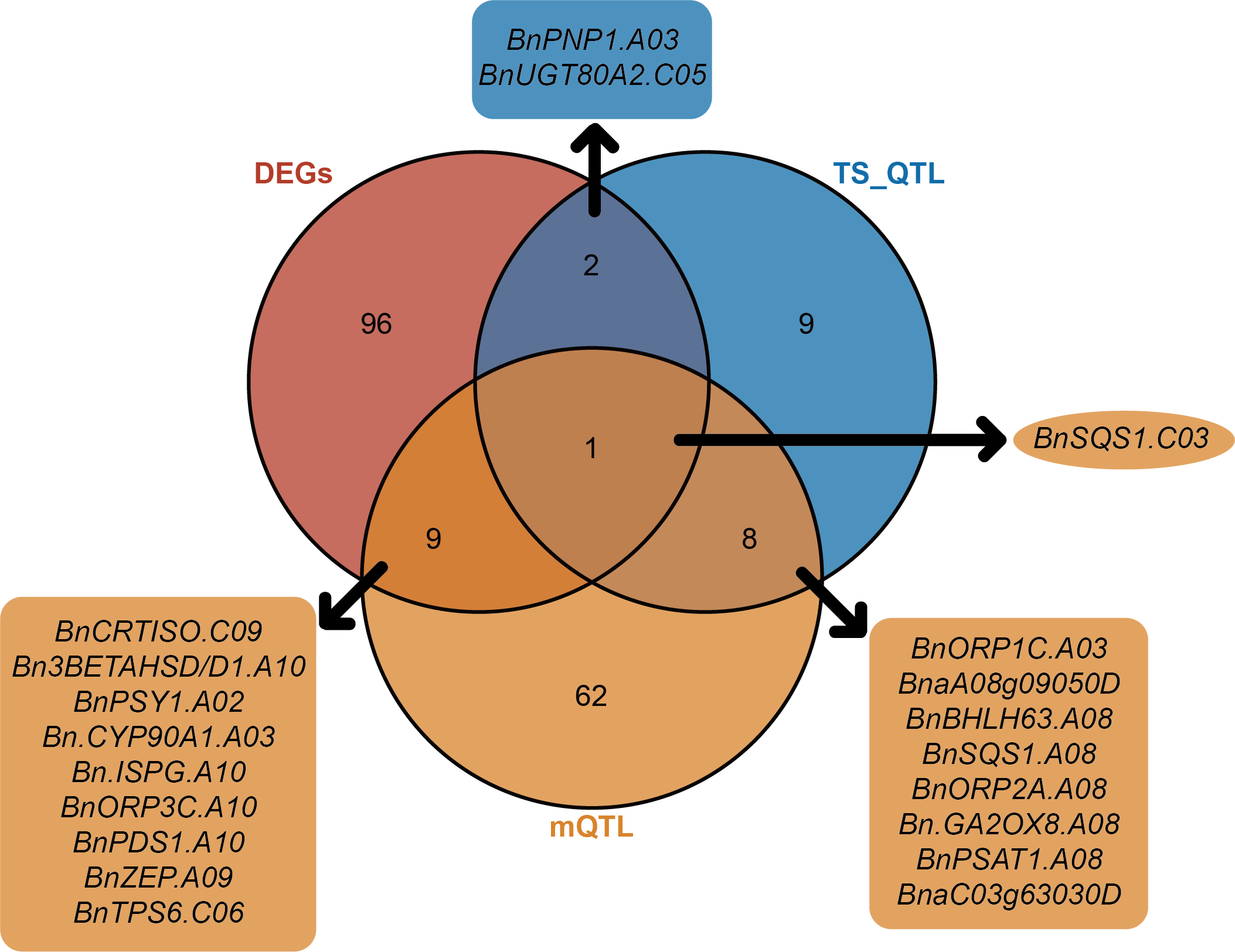


**Figure S9.** Venn diagram that represents the gene correlations among sterol-related DEGs, TS QTL, and mQTL intervals. Genes that overlap in these regions are indicated by arrows.


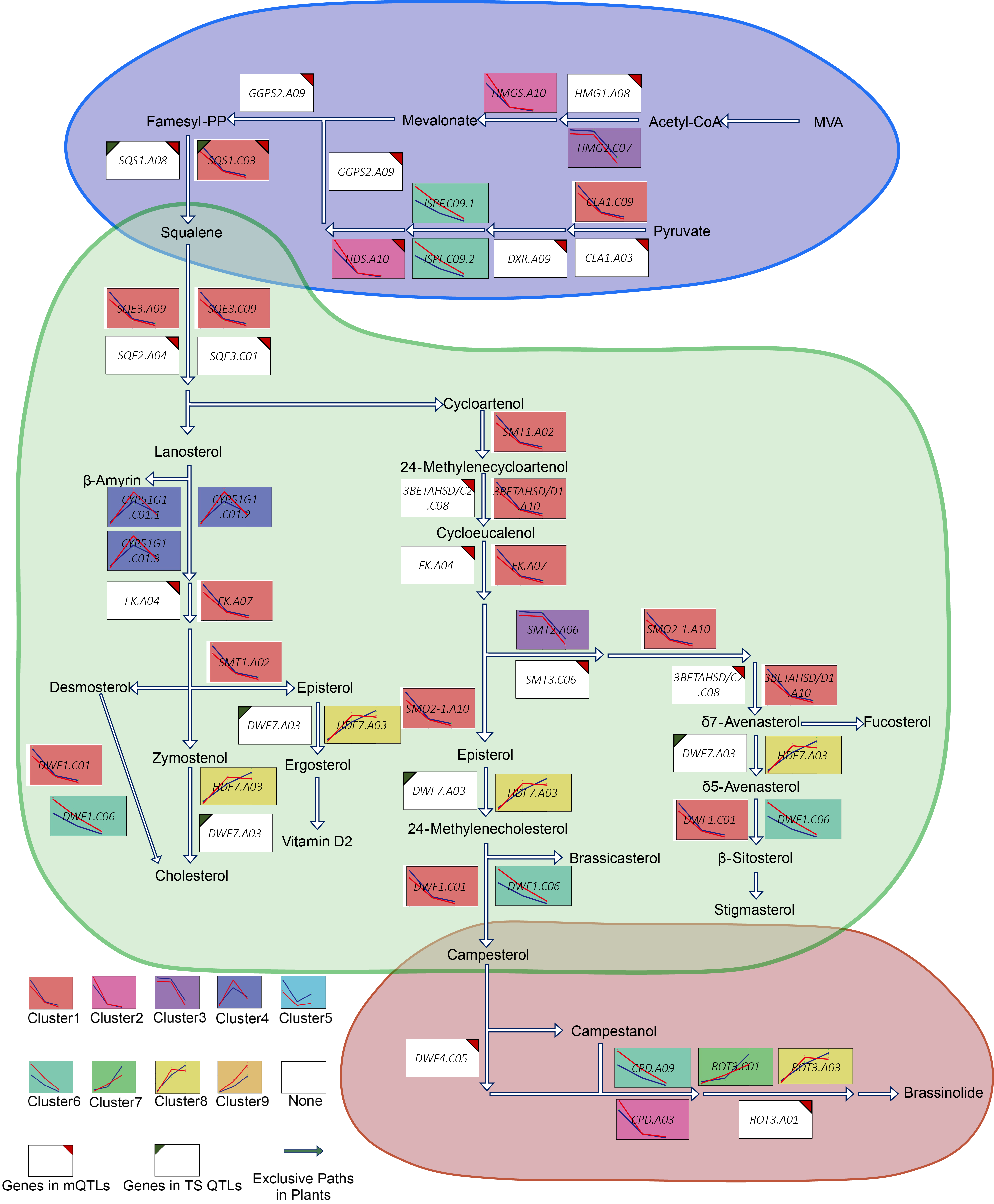


**Figure S10.** Potential regulatory model of sterol content variation in *B*. *napus*. Candidate genes from different sources are labeled with different colors, and multiple colors are used if different sources are available at the same time. Candidate genes are divided into three regions according to different biological processes and are boxed with different colored dashed lines.


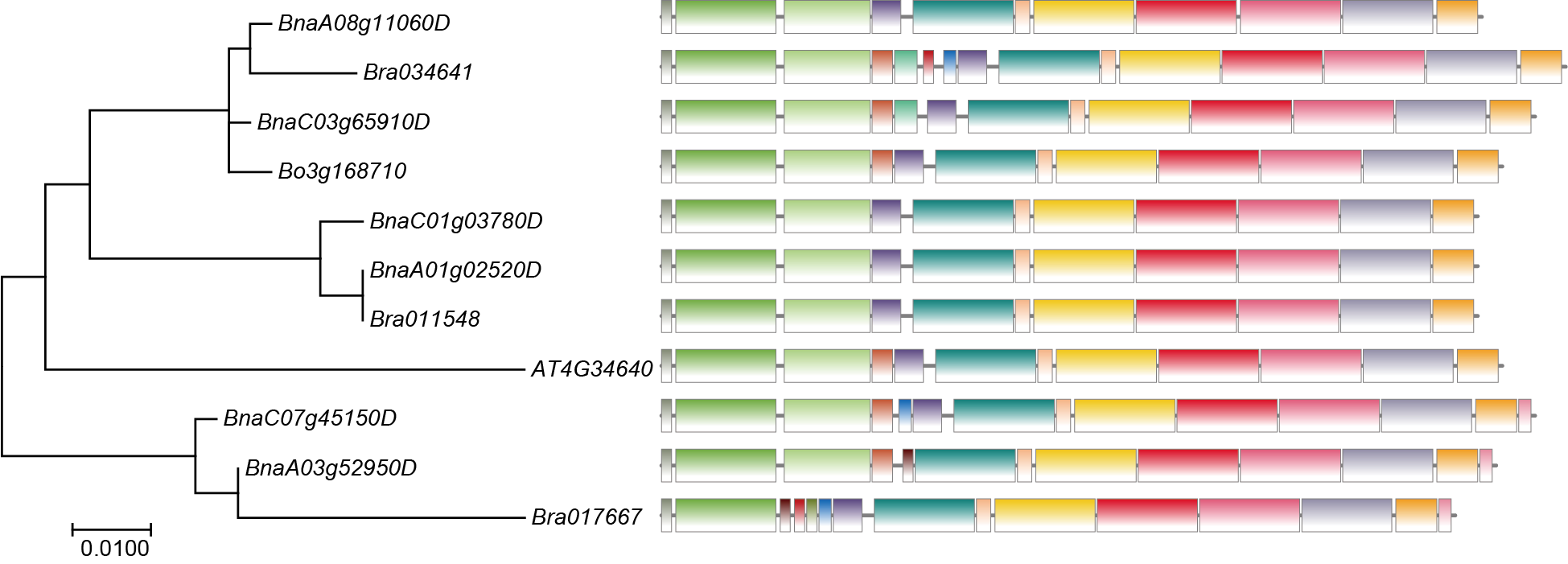


**Figure S11.** Phylogenetic analysis of the amino acid sequences of SQS1 homologous genes and motif analysis. On the left is the phylogenetic tree generated by amino acid sequence alignment, and on the right are the motifs on the amino acid sequences, with different colored rectangles indicating different motifs.


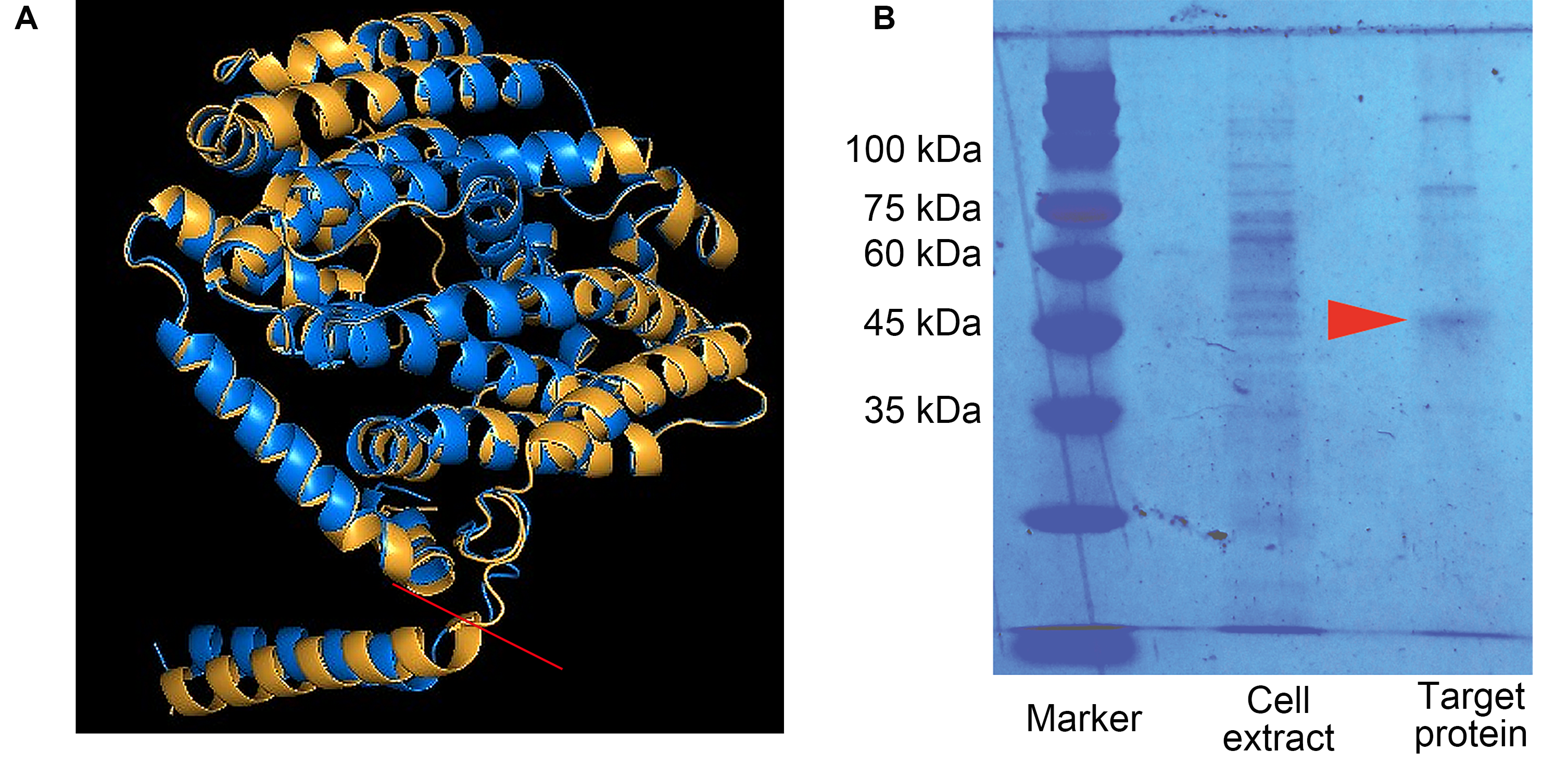


**Figure S12.** (A)Alignment of the predicted protein structures of *BnSQS1.C03* and *AtSQS1*, with the red line indicating the starting amino acid of the transmembrane region to be deleted. (B) SDS-PAGE validation results of purified protein, from left to right: marker, protein supernatant, purified protein. The target protein is marked with a red arrow.


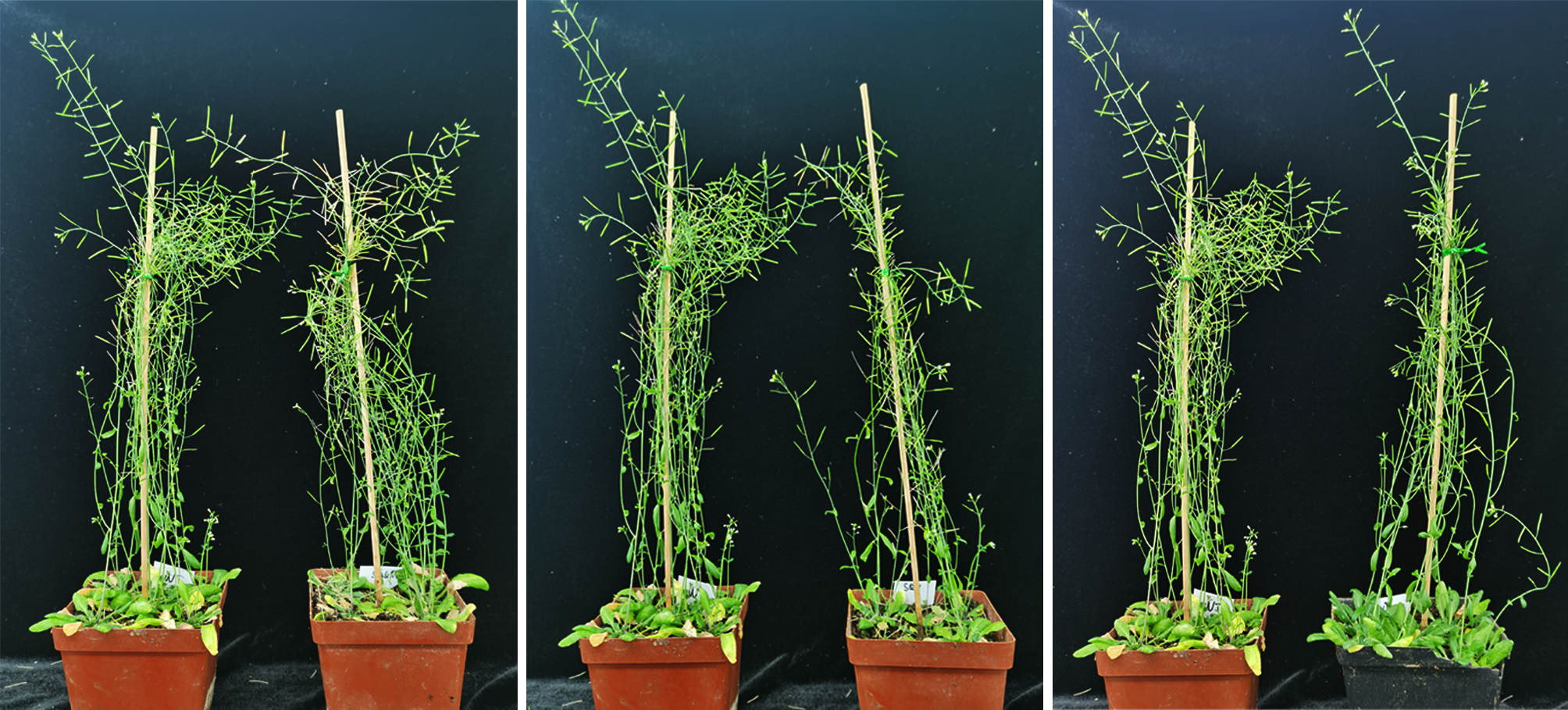


**Figure S13.** The *A. thaliana* plants in these three images, from left to right, are Col-0 vs Col-0^BnSQS1.C03^, Col-0 vs *atsqs1*, and Col-0 vs *atsqs1*^BnSQS1^.
